# Bispecific antibodies tethering innate receptors induce human tolerant-dendritic cells and regulatory T cells

**DOI:** 10.3389/fimmu.2024.1369117

**Published:** 2024-03-26

**Authors:** Lucille Lamendour, Mäelle Gilotin, Nora Deluce-Kakwata Nkor, Zineb Lakhrif, Daniel Meley, Anne Poupon, Thibaut Laboute, Anne di Tommaso, Jean-Jacques Pin, Denis Mulleman, Guillaume Le Mélédo, Nicolas Aubrey, Hervé Watier, Florence Velge-Roussel

**Affiliations:** ^1^EA7501, Groupe Innovation et Ciblage Cellulaire, Team Fc Récepteurs, Anticorps et MicroEnvironnement (FRAME), Université de Tours, Tours, France; ^2^Infectiologie et Santé Publique (ISP) UMR 1282, INRAE, Team BioMAP, Université de Tours, Tours, France; ^3^institut de recherche pour l’agriculture, l’alimentation et ’environnement (INRAE) UMR 0085, centre de recherche scientifique (CNRS) UMR 7247, Physiologie de la Reproduction et des Comportements, Université de Tours, Tours, France; ^4^MAbSilico, Tours, France; ^5^Dendritics, Lyon, France; ^6^Service de Rhumatologie, Centre Hospitalo-Universitaire (CHRU) de Tours, Tours, France

**Keywords:** human dendritic cell, innate receptors, type C lectin, TLR2, bispecific antibody, tolerant dendritic cell, interleukine-10, TGF-β1

## Abstract

There is an urgent need for alternative therapies targeting human dendritic cells (DCs) that could reverse inflammatory syndromes in many autoimmune and inflammatory diseases and organ transplantations. Here, we describe a bispecific antibody (bsAb) strategy tethering two pathogen-recognition receptors at the surface of human DCs. This cross-linking switches DCs into a tolerant profile able to induce regulatory T-cell differentiation. The bsAbs, not parental Abs, induced interleukin 10 and transforming growth factor β1 secretion in monocyte-derived DCs and human peripheral blood mononuclear cells. In addition, they induced interleukin 10 secretion by synovial fluid cells in rheumatoid arthritis and gout patients. This concept of bsAb-induced tethering of surface pathogen-recognition receptors switching cell properties opens a new therapeutic avenue for controlling inflammation and restoring immune tolerance.

## 1 Introduction

Despite the interest in antibody (Ab)-based therapeutics to control inflammatory diseases, few such therapies have been or are currently being developed to target human dendritic cells (DCs). This lack of development could be explained by the fact that nearly all of the Abs, including CTLA4-Fc, anti-CD40, and anti-OX40L Abs, are antagonists of costimulatory molecules, which, by principle, induce strong immunosuppression rather than “gently” regulate human DCs (1–3). To our knowledge, no bispecific Ab (bsAb) drug product with a mechanism of action targeting two antigens on DCs to induce their inhibition is being developed (4, 5).

As antigen-presenting cells, DCs play an important role in the immune response because they are able to control the balance between immune tolerance and the immune response (6). Two DC subsets, which are present in human blood and almost all lymphoid and nonlymphoid tissues, are conventional DCs (cDCs) and plasmacytoid DCs (pDCs) (7). Each subset is characterized by both surface expression markers and functional properties. The cDCs are identified by the expression of both major histocompatibility complex class II (MHC class II) and cCD11c markers. In humans, they are divided into two subsets, cDC1 and cDC2, according to their expression of CD141 (blood DC antigen 3 [BDCA3]) and DC1c (BDCA1), respectively (7). Each of the cDC subtypes has specific properties: the cDC1 subtype has a great capacity for cross-presentation and cytotoxic T-lymphocyte induction, whereas the cDC2 subtype is better at promoting T helper 2 (Th2) and Th17 cells. The pDCs are critical for producing type I interferon during viral infections. They are characterized by their expression of CD123, CD303 (BDAC2), MHC class II, and CD304 (BDCA4) (8). Monocyte-derived DCs (moDCs) form a distinct population of DCs that appear and differentiate *in situ* at inflammation sites. They express MHC class II, CD11c, CD1c (BDCA1), CD1a, CD206 (mannose receptor), and CD209 (9). Each subset has specific pathogen recognition receptors (PRRs) that allow them to identify danger signals and initiate their maturation. These PPRs include C-type lectin receptors (CLRs) and Toll-like receptors (TLRs), which are the most abundant PPRs on the DC surface (10).

The most astonishing property of DCs is their capacity to control the balance of immune responses (11). Tolerant DCs (tolDCs) represent the only effective way to induce regulatory T cells (Tregs), which are the main actors in this homeostasis. Several molecules have been used to induce *in vitro* tolDCs, which induce DC semimature profiles and tolerant cytokine secretions (12). Relevant mouse models treated with these molecules showed an improvement in skin and organ graft features (13). Likewise, adoptive transfer of *ex vivo*-generated tolDCs induced a better outcome in arthritis (14). In humans, clinical trials of tolDC adoptive transfer did not confer real patient benefits. The possibility of inducing *in situ* tolDCs in humans may be at hand.

CLRs, as innate immunity receptors, are used to signal hazards but are also found in immune regulation induction (10). CD209 (DC-SIGN), in association with TLR4, promoted *Mycobacterium tuberculosis* infection by inducing local immune tolerance (15). Moreover, CD367 (DC immunoreceptor [DCIR]) has been repeatedly mentioned as a therapeutic target because it regulates immune tolerance to self and because autoimmune diseases developed in DCIR^KO^ mouse models (16). The activated TLR2 pathway can induce interleukin 10 (IL-10)-secreting DCs and is used by different pathogens to jeopardize immune system efficiency (17, 18). In addition to molecules that activate immune response inhibitory pathways, pathogens have deployed an alternative strategy with molecules capable of bridging receptors on the cell surface, as exemplified by yeasts, which produce zymosan (zym) (17, 19, 20). This compound crosslinks TLR2 and dectin-1, a PRR of the CLR family (17, 20). TLR2 is the only ambivalent TLR, able to induce inflammation or immune tolerance depending on the context. By inducing TLR2 cross-linking with dectin-1, zym paradoxically rendered DCs tolerant and able to induce Tregs (17, 21). Although zymosan is sometimes used clinically, it remains a complex mixture that is difficult to control and only activates dectin-1.

Here, we generated bsAbs tethering TLR2 and two other CLRs that switch moDCs to a tolerant profile to be able to differentiate Tregs. These molecules seemed to be able to reverse the inflammatory profile of synovial fluid (SF) cells in gout or rheumatoid arthritis (RA) patients. This strategy could constitute a new avenue to render *in situ* DCs tolerant.

## 2 Materials and methods

### 2.1 Antibody expression and purification

The OPN-305 monoclonal antibody is a fully humanized antibody derived from the OPN-301 monoclonal antibody, a murine IgG1 anti-TLR2 antibody (mouse Toll-like receptor 2 (TLR2) antibody, clone T2.5; http://www.faqs.org/patents/app/20120164159) (22). Murine antihuman CD209 (DDX0208, clone 120C11) and antihuman DCIR (clone 8H8 103.3) antibodies were developed by Dendritics (Lyon, France). The anti-TLR2 scFv was combined with either the anti-DC-SIGN or anti-DCIR scFvs to form the tandem scFvs. The tandem scFvs were designed as follows: VL_[TLR2]_-(G_4_S)_3_-VH_[TLR2]_-(G_4_S)*_x_*-VH_[DC-SIGN or DCIR]_-(G_4_S)_3_-VL_[DC-SIGN or DCIR]_-G_3_AS-HHHHHH. Bic03 corresponds to anti-TLR2*x* anti-CD209 with a long linker (*x* = 3). Bic05 corresponds to the anti-TLR2*x* anti-DCIR with a short linker (*x* = 1). The VL sequence of anti-TLR2 was mutated so that it could interact with the L protein (23). For this purpose, the VL_[TLR2]_ sequence was modified at residues 9–22 and 90 according to IMGT nomenclature (9–22: ATLSLSPGERATLS-SSLSASVGDRVTIT and K90T).

The genes were then synthesized and cloned into the pcDNA3.4 plasmid by GeneArt (ThermoFisher Scientific, Waltham, MA, USA). Tandem scFvs were produced by transitory transfection of ExpiCHO™ cells with the max titer protocol as described in ThermoFisher’s manufacturer’s protocols. Briefly, cells were grown in ExpiCHO™ Expression Medium and maintained in a humidified incubator with 8% CO_2_ at 37°C under shaking. Prior to transfection (Day −1), cell concentration was adjusted at 4·10^6^ viable cells/mL and incubated overnight in culture-grown conditions. On the next day (Day 0), the cell culture was diluted to 6·10^6^ cells/mL and transfected by 0.8 µg/mL of plasmid encoding Bic03 or Bic05, previously mixed with ExpiFectamine CHO reagent. The expression enhancer was added at 18 h and left until 22 h posttransfection, and the flask was placed at 32°C with 5% of CO_2_. An additional expression feed was added on day 5, and cells were harvested around 10 days posttransfection. Cell viability was measured with CytoSMART™ (Corning, NY 14831 USA) and centrifuged at 10,000×*g* for 10 min. Clarified supernatants were stored at −20°C until purification.

For purification of antibodies, samples were deep-frozen, centrifuged at 10,000×*g* for 20 min, and passed over a 0.22-µm filter. All supernatants were passed over HiScreen™ Capto™ L column (Cytiva, Velizy-Villacoublay, France) equilibrated with PBS buffer (2.7 mM of KCl, 0.10 M of NaCl, 2 mM of KH_2_PO_4_, 8 mM of Na_2_HPO_4_, pH 7.4) in the ÄKTA pure protein purification system. Tandem scFv was eluted by a linear pH gradient in 0.1 M glycine buffer running from pH 6 to pH 2, and the buffer was removed by a desalting column in PBS. The antibodies are concentrated by Amicon (Merk Millipore, Darmstadt, Germany) and filtered (0.2 µM—Merck Millipore, Darmstadt, Germany). Antibody concentration was determined with a UV detector at 280 nm. Tandem scFvs molecular mass and molar extinction coefficient data were all generated by the Protparam tool from http://web.expasy.org/protparam/. Integrities of all purified proteins were assessed by sodium dodecyl sulfate-polyacrylamide gel electrophoresis (SDS-PAGE) on homogeneous 10% polyacrylamide gel under reducing or nonreducing conditions. Samples were all loaded at 0.5 µg for Coomassie blue staining. ProSieve QuadColor Protein Markers (Lonza, Fribourg, Switzerland) were used. Each tandem and scFv were centrifuged at 15,000×*g* at 4°C for 20 min before use.

Endotoxin levels in the purified antibodies were determined by the Limulus amebocyte lysate test according to the manufacturer’s instructions (Thermo Scientific™ Pierce™ LAL Chromogenic Endotoxin Quantitation Kit, Thermo Scientific, Illkirch, France). Antibody concentration measures were performed using the BCA method (BCA Protein Assay Kit, Sigma-Aldrich, Germany) and optical density at 208 nm by Nanodrop (Thermo Scientific™ NanoDrop™ One/One^C^, Illkirch, France).

### 2.2 Human monocyte-derived dendritic cells

Cytapheresis products were obtained from the Centre Atlantic Transfusion Department (EFS-CA, Tours, France) according to the research agreements CPDL-PLER 2016 004 and CDPL-PLER 2019 188. They were issued from the healthy adult volunteers who had given their written informed consent, and the EFS ethics committee approved the procedure.

To prepare peripheral blood mononuclear cells (PBMCs), whole blood was diluted one-half with PBS. Density gradient separation of blood involved using Lymphoprep (Eurobio, Les Ulis, France). Tubes were centrifuged at 450×*g* for 25 min at 25°C, then cell layers (buffy coat) were immediately collected and transferred to 50 mL conical tubes, resuspended with PBS, and centrifuged at 300×*g* for 10 min at 25°C. Monocytes were then purified from PBMCs by a positive selection using CD14 microbeads (Miltenyi Biotec, Bergisch Gladbach, Germany) (> 90% purity). For immature monocyte-derived DCs (moDCs), monocytes were differentiated for 6 days in RPMI 1640 medium (Dominique Dutscher, Bernolscheim - France) medium supplemented with 10% FCS (Dominique Dutscher, France), 66 ng/mL of granulocyte-macrophage colony-stimulating factor (GM-CSF), and 25 ng/mL of IL-4 (Miltenyi Biotec, Bergisch Gladbach, Germany). On day 6, cells were collected, and flow cytometry analysis was performed for moDC qualification.

MoDCs were treated with different maturation agents, such as zymosan from *Saccharomyces cerevisiae* (Sigma-Aldrich, St. Louis, MO, USA) at 5 μg/mL, or antibodies: neutralizing anti-TLR2 antibody (Bio-Techne, Minneapolis, MN, USA), anti-CD209 (clone 120C11.01, Dendritics, Lyon, France), anti-DCIR (clone 108H8.3, Dendritic, Lyon, France), the corresponding scFvs (αTLR2, αCD209, αDCIR), or the bsAbs (Bic03, Bic05) at different concentrations at 37°C in a 5% CO_2_ atmosphere. The DC maturation and secretion of cytokines were assessed after 48 h by flow cytometry and ELISA.

### 2.3 PBMC cultures

After isolation, PBMC (1·10^6^ cells/mL) was placed in culture in the presence of zym (5 μg/mL), Bic03 at different concentrations, or controls scFvs for 48 h. The percentage of CD4^+^/CD25^+^/CTLA-4^+^/PD-1^+^ (Miltenyi Biotec, Bergisch Gladbach, Germany) was then determined by flow cytometry. Longer PBMC cultures were performed with Bic05, zym (5 μg/mL), or scFvs with 1·10^6^ cells/mL for 2, 6, and 9 days. At day 5, IL-2 was added at 100 UI/mL (Miltenyi Biotec, Bergisch Gladbach, Germany) to all culture conditions. Within these PBMC cultures, the phenotypic profiles of CD4^+^ regulatory T cells and DC subsets were analyzed at D2, D6, and D9 by flow cytometry according to previously described panels.

### 2.4 Human T cells

The T lymphocytes were obtained from PBMCs by positive, then negative, bead selection to obtain the CD4^+^/CD25^−^ fraction (Miltenyi Biotech, Bergisch Gladbach, Germany). For T-cell differentiation, 1·10^6^ CD4^+^ T cells were cultured with 1·10^5^ allogeneic DC (10:1, T:DC). After 10 days, primed T cells were collected and purified using CD4 microbeads (Miltenyi Biotech, Bergisch Gladbach, Germany). For recall response proliferation, primed CD4^+^ T cells were stained with Cell Proliferation Dye eFluor R 670 (eBioscience, CA, USA, Villebon sur Yvette, France) and plated with DCs treated with Bics from the same donor used for priming (T:DC, 10:1). After 2, 4, and 6 days, the percentages of divided responder T cells were calculated by proliferation dye dilution by flow cytometry, and the percentage of the CD4^+^/CD127*^low^*/CD25^+^/Foxp3^+^/IL-10^+^ population was evaluated by flow cytometry.

For the evaluation of the inhibition properties of Bic05-PBMC populations, allogenic CD4^+^/CD25^−^ were stained with Cell Proliferation Dye eFluor R 670 (eBioscience, CA, USA) and then stimulated with CD3/C28 Dynabeads (Corning, NY, USA) in the presence of D9-cultured PBMC cells in a ratio 1:1. The CD4^+^ proliferation was evaluated at D5 and D6 by flow cytometry.

### 2.5 Human clinical study

Ethics approval and consent to participate: this study was a noninterventional clinical trial (ID RCB: 2017-A02678-45) and approved by the institutional review board—”Comité de Protection des Personnes - Ile de France VIII” (CPP: 17 11 76). The patients included received information on the use of their samples and medical data for research purposes. The present study was registered at ClinicalTrials.gov (NCT03416543). Patient demographics for the clinical trial analyzed in this study are provided in **Supplementary Table 1**. All patients gave informed consent, and the studies were approved by their respective ethical review committees.

### 2.6 Synovial sample preparation and purification

Aspiration of SF was performed in each patient after strict aseptic techniques. Samples were stored at 4°C until they were analyzed in the laboratory. SF dilutions (one-third) were performed in RPMI 1640 (Dominique Dutcher, Brumath, France) and centrifuged at 700×*g* for 10 min. Cell pellets were counted in the presence of trypan blue. For flow cytometry, cells were washed with PBS (Gibco, ThermoFisher, Waltham, MA, USA) containing 1% SVF and 0.01% N_3_Na in 96-well plates. Next, cells (1·10^6^/100 μL) were stained for 30 min at 4°C with the appropriate antihuman panel at the appropriate concentration or with the relevant isotypes (see “Flow cytometry analysis” paragraph).

The cells (1·10^6^/mL) from SF were maintained in culture in the presence of Bic03, Bic05, Zymosan (5 μg/mL), or medium (RPMI 1640, 10% FCS, 1% glutamine) as control at 37°C for 48 h. After 48 h, samples were centrifuged at 300×*g*. Culture supernatants were then stored at 20°C until ELISA analysis.

### 2.7 Epitope prediction

Epitope prediction was made using the software MAbTope (24). The 3D structures used for the targets were 2Z7X for TLR2, 1XPH for DC-SIGN, and 5B1X for DCIR. The anti-TLR2 scFv was modeled by homology, using 7Q4Q (VH) and 7MW5 (VL) as templates. The anti-DC-SIGN scFv was modeled by homology, using 7KYO (VH) and 4S2S (VL) as templates. The anti-DCIR was modeled by homology, using 3WII (VH) and 1A6V (VL) as templates. All models were built using MODELLER (25).

### 2.8 Flow cytometry

Flow cytometry was performed on a Cytoflex S flow cytometer (Beckman Coulter, Brea, CA, USA) with FlowLogic software (Miltenyi Biotec, Bergisch Gladbach, Germany). Results were expressed as the ratio of mean of fluorescence (MFI) of the marker to the MFI of the isotype control and referred to as MFI ratio, either as the MFI of positive cells minus the isotypic control MFI or referred to as MFI.

Cells (1·10^6^–2·10^5^/100 μL) were stained for 30 min at 4°C in cold PBS containing 1% SVF and 0.01% NaN_3_ (Sigma-Aldrich, St. Louis, MO, USA) in the presence of appropriate VIO-Dyes (ThermoFisher, Waltham, MA, USA) with the following antihuman antibodies at the appropriate concentration or with the relevant isotypes according to the following panels:

For DC qualification at D6 of CD14^+^ culture: antihuman CD45-PEvio770, CD209-PE, and CD14-PercP (Miltenyi Biotec, Bergisch Gladbach, Germany).

For DC maturation: CD83-FITC, CD86-PE, CD25-APC, PD-L1-FITC (BD Biosciences, San Jose, CA, USA), CD14-PE, ILT3-PE-Cy7, ILT4-PE, (Beckman Coulter, Brea, CA, USA), HLA-DR–Percp (Miltenyi Biotec, Bergisch Gladbach, Germany), IL-10 receptor alpha-PE, IL-10 receptor beta-FITC (R&D Systems, Minneapolis, MN, USA) in three different panels.

In PBMC culture: for CD4^+^ cell identification, the following antibody pattern were used: CD4-viogreen, CD25-FITC, CD152-PE-CF594, Lap-APC (Lap (TGF-β1), IL-10-PE, CD73-APC750, CD127-PEcy7 (BD Biosciences, Minneapolis, MN, USA), Foxp3-Bv421 (BioLegend, San Diego, CA, USA), and PD1-PE-Cy7 (R&D).

For DC subset identification, the following antibody patterns were used:

HLA-DR-FITC, CD123-PE, BDCA-2-PEvio770, CD11c-PE, CD1c-PEvio700, CD141-PEvio700, IL-10-APC, (Miltenyi Biotec, Bergisch Gladbach, Germany), and TGFb1-Bv421 (BioLegend, San Diego, CA, USA).

- In synovial cells: CD45-PEvio770, CD3-PE/CD56-FITC, CD14-PE/CD19-FITC, and CD66b-PE (Miltenyi Biotec, Bergisch Gladbach, Germany).

For the DC subset identification in synovial cells, the following panel was used: HLA-DR-FITC, CD123-PE, CD303-PEVio700, CD11c-PE, CD1c-PEvio770, and CD141-PE-vio770. All cells were labeled by TLR2-APC, DCIR-APCvio770, and CD209-PE-Vio700 (Miltenyi Biotec, Bergisch Gladbach, Germany).

The cells were then washed once using cold PBS containing 1% SVF and 0.01% NaN_3_, and viable cells were then analyzed by flow cytometry.

### 2.9 BsAb binding to moDCs

MoDCs (1·10^6^ cells/100μL) were incubated with different concentrations of Bic03 or Bic05 at 37°C for at least 1 h. The cells were washed two times with cold PBS containing 1% SVF and 0.01% NaN_3_ and incubated with anti-histidine-PE (R&D Systems, Minneapolis, MN, USA) or anti-histidine–APC antibody (Miltenyi Biotech, Bergisch Gladbach, Germany) for 1 h. For binding inhibition experiments, anti-TLR2 (clone T2.5, Invivogen Toulouse, France), anti-CD209, anti-DCIR antibodies (Dendritics, Lyon, France) in the presence of Fc block (Miltenyi Biotech, Bergisch Gladbach, Germany) or anti-TLR2, anti-CD209, anti-DCIR corresponding scFvs were incubated at least 30 min (Bic05) or 1 h (Bic03) before adding Bic03 or Bic05. The binding of both bsAbs was demonstrated thanks anti-Hist-PE/-APC antibody as described above.

### 2.10 ELISA/LegendPlex

MoDCs or PBMC (1·10^6^/mL) were stimulated for 48 h by medium, Zymosan (5 μg/mL), Bic03 or Bic05. The culture supernatants were collected, and centrifuged at 10,000×*g* for 10 min at 4°C, and then IL-10, IL-12-p70, TNF-α, and IFN-γ ELISA measurement was performed on culture supernatants of each condition according to the manufacturer’s instructions (ThermoFisher, Waltham, MA, USA). Data were expressed as means ± SD of donors. LegendPlex™ was used to measure the secretion of cytokines from PBMC cells (IL-4, IL-2, CXCL10 (IP-10), IL-1β, TNF-α, CCL2 (MCP-1), IL-17A, IL-6, IL-10, IFN-γ, IL-12p70, CXCL8 (IL-8), and Free Active TGF-β1. The protocol was performed per the manufacturer’s directions. Briefly, detection beads were sonicated and incubated with media from purified cells. Beads bound to target cytokines were then washed, incubated with detection antibodies, and washed again, and the cytokine concentrations were then determined through flow cytometric analysis and a standard curve. The Cytoflex S (Beckman Coulter) was used for data acquisition, and accompanying LegendPlex™ software was used for analysis.

### 2.11 Clinical study design

Adult patients with known or newly diagnosed RA, primitive OA, or gout disease who presented with mono-, oligo-, or polyarthritis and underwent aspiration of synovial fluid were included. Patients with microcrystalline arthritis other than gout, spondylo-arthritis, septic arthritis, or receiving therapeutic antibodies were excluded. Patient’s consent forms were collected before each inclusion after they were given loyal and complete information. The diagnosis of RA was established using the ACR/EULAR criteria 2010. Gout disease was diagnosed in the presence of multiple criteria, such as tophus, monosodium urate crystals found in the SF, and hyperuricemia.

### 2.12 Statistical analysis

All data are presented as mean ± SD unless otherwise stated. Statistical and graphical analyses were made using GraphPad Prism 8 (GraphPad Software, San Diego, CA, USA). Statistical significance was determined by a one-way ANOVA to compare differences among multiple groups. *p* < 0.05 was considered statistically significant.

## 3 Results

### 3.1 Engineering and characterization of bsAbs

To mimic zym, we designed a bsAb to crosslink TLR2 and a CLR on the DC surface. We selected a murine monoclonal Ab (mAb) against CD209 (DC-SIGN) that does not activate human moDCs, induce their IL-10 secretion, or inhibit zym-induced IL-10 secretion (**Supplementary Figure 1A**). All bsAbs and single-chain fragment variables (scFvs) were analyzed by SDS-PAGE, size-exclusion chromatography, and mass spectrometry (**Supplementary Figures 2B–D**). The sample productions were checked for endotoxin contamination (**Supplementary Figure 2E**). We also evaluated the presence and expression of bsAb targets on moDCs, showing a low level of TLR2 and a high level of CD209 (**Supplementary Figure 3A**).

The mAbs were directed against different domains of CD209 leading to different DC maturation types (26). We determined the putative epitope of our anti-CD209 Ab by applying a new and robust *in silico* method (24). The epitope was located in the carbohydrate recognition domain (**Supplementary Figure 4A**), a location that does not inhibit endocytosis (26). We also selected an anti-TLR2 mAb that had been designed under an scFv format (22) and whose epitope was predicted to be in the 152-169 aa region of TLR2 (**Supplementary Figure 4B**). The anti-CD209 mAb was derived under the same scFv format and attached to the anti-TLR2 scFv via a (G4S)_3_ linker (**Supplementary Figure 2A**). Such a construct was named “bispecific anti-C-lectin” (Bic). None of the scFvs alone could induce moDC IL-10 secretion (**Supplementary Figure 1B**).

### 3.2 Bic03 (αTLR2xαCD209*)* binding on moDCs modifies cell profiles

**Figure 1:**
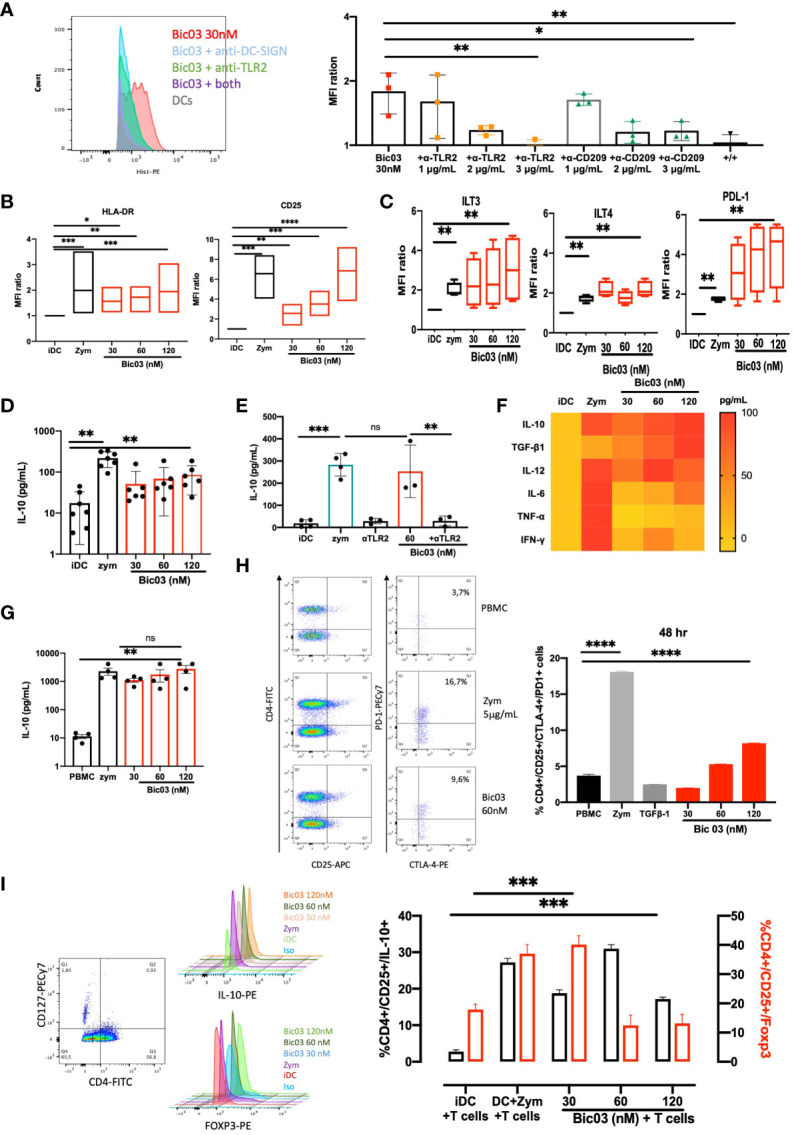
Functional properties of αTLR2xαCD209 (Bic03). **(A)** MoDCs were incubated with Bic03 at 37°C for 1 h, and Bic binding was revealed with an anti-His mAb on flow cytometry. Bic03 binding inhibition involved adding anti-CD209 and anti-TLR2 scFvs at increasing doses from 1 to 3 μg/mL (*n* = 3, ^*^p < 0.04, ^**^p < 0.01). **(B)** MoDC maturation status according to expression of HLA-DR (*n* = 7, ^*^p < 0.01, ^**^p < 0.001, ^***^p < 0.0006) and CD25 (*n* = 7, ^**^p < 0.02, ^***^p < 0.0006, ^****^p < 0.0001) receptors on flow cytometry. Box-and-whisker plots indicate median and minimum–maximum. **(C)** Surface expression of ILT3, PD-L1, and ITL4 on moDCs in the presence of Bic03 (60 nM) (*n* = 5, ^**^p < 0.001). **(D)** IL-10 secretion by moDCs treated with Bic03 for 48 h measured by ELISA (*n* = 6, ^**^p < 0.001). **(E)** IL-10 secretion inhibition by moDCs with scFv anti-TLR2 mAb (*n* = 4, ^***^p < 0.0001, ^**^p < 0.001). **(F)** Cytokine secretion profiles of moDCs treated with Bic03 after 48 h as a heatmap (*n* = 8). **(G)** PBMC secretion of IL-10 after 48-h treatment with Bic03 (*n* = 4, ^**^p < 0.001). **(H)** Dot plots and proportion of CD4^+^/CD25^+^/CTLA-4^+^/PD-1^+^ cells after 48-h culture of PBMCs and dot plots with Bic03 measured by flow cytometry (one of four experiments; ^****^p < 0.0001). **(I)** Dot plots and proportion of CD4^+^/CD127^−^/CD25^+^ IL-10^+^ (black) or Foxp3^+^ (red) cells in MLR in 9-day culture (*n* = 3, ^***^p < 0.0003). scFv, single-chain fragment variable; MLR, mixed lymphocyte reaction. ns, not significant.

The binding of Bic03 to donor moDCs was dose-dependent (**Supplementary Figure 5A**) and could be inhibited by both anti-TLR2 and/or anti-CD209 scFvs (**Figure 1A**). MoDC treatment with Bic03 induced a semimature phenotype different from that with zym (**Figure 1B**; **Supplementary Figure 6A**) as well as the expression of regulatory molecules such as immunoglobulin-like transcript (ILT3; CD85k) or ILT4 (CD85d) and programmed death-ligand 1 (PD-L1) (CD274) (**Figure 1C**). Bic03 also induced the expression of CCR7 to the same level as with zym (**Supplementary Figure 7A**). In parallel, Bic03 dose-dependently induced IL-10 secretion by moDCs (**Figure 1D**), but the corresponding scFvs did not, even in combination (**Supplementary Figure 1B**). This effect could be inhibited by the anti-TLR2 scFv alone (**Figure 1E**). However, this semimature profile and the amount of IL-10 secretion were not sufficient to consider Bic03-treated DCs as tolDCs (27), and we further explored cytokine secretion panels. The cytokine secretion was clearly different from that obtained with zym, with a higher level of TGF-β1 and lower levels of IFN-γ, IL-6, and TNF-α (**Figure 1F**).

### 3.3 PBMC cultures with Bic03 induce regulatory populations

To further analyze the effect of Bic03 on human cells, donor PBMCs were treated for 48 h with different concentrations of Bic03. We found strong IL-10 secretion (**Figure 1G**) and dose-dependent induction of CD4^+^/CD127^low^/CD25^+^/CTLA-4^+^/PD-1^+^ cells after 48 h (**Figure 1H**). Moreover, mixed lymphocyte reaction (MLR) 6-day cultures of naïve allogenic CD4^+/^CD25^−^ cells and Bic03-treated moDCs increased CD4^+^/CD127^low^/CD25^+^/Foxp3^+^ and/IL-10^+^ Treg proportion, which disappeared at higher Bic03 concentrations (**Figure 1I**). Thus, Bic03 seemed able to induce tolerogenic moDCs and phenotypical Treg populations.

### 3.4 Bic05 (αTLR2xαDCIR) shows comparable properties as Bic03

**Figure 2:**
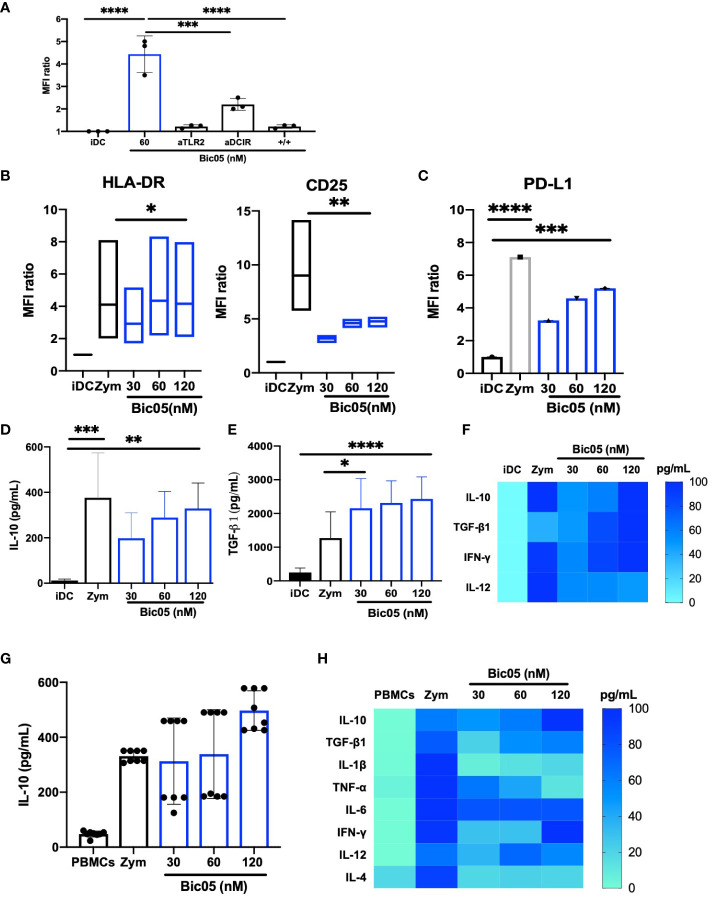
Functional properties of Bic05 (aTLR2xaDCIR). **(A)** Quantification of binding inhibition of Bic05 on moDCs by anti-DCIR or anti-TLR2 scFvs on flow cytometry on MFI ratio (*n* = 3, ^****^p <0.0001, ^***^p < 0.0003). **(B)** MoDC maturation status by expression of HLA-DR and CD25 receptors on flow cytometry. Box-and-whisker plots indicate median and minimum–maximum (*n* = 6, ^*^p < 0.01, ^**^p < 0.001). **(C)** Bic05-treated moDC expression of PDL-1 (*n* = 5, ^****^p < 0.0001, ^***^p < 0.0009). Bic05-treated moDC secretion **(D)** of IL-10 (*n* = 4, ^***^p < 0.0002, ^**^p < 0.003) and **(E)** TGF-β1 (*n* = 6, ^***^p < 0.0006, ^*^p < 0.03). **(F)** Heatmap of cytokine secretion by moDCs (*n* = 7). **(G)** Bic05-treated PBMC secretion of IL-10 (*n* = 5, ^**^p < 0.001). **(H)** Heatmap of cytokine secretion by PBMCs at 48 h (*n* = 5). MFI ratio, mean of fluorescence ratio.

Bic03 being difficult to produce and to extend our observations, we designed another Bic targeting TLR2 and a CLR, namely DCIR (CD367), which is an immunoreceptor tyrosine-based inhibitory motif-dependent CLR, unlike CD209, which is an immunoreceptor tyrosine-based activation motif-dependent CLR. As for Bic03, we selected a murine mAb against human DCIR that could not activate moDCs (**Supplementary Figure 1A**) and also recognized the carbohydrate recognition domain (**Supplementary Figure 4C**). As compared with Bic03, for Bic05, the scFv derived from the mAb was associated with the anti-TLR2 scFv by a shorter linker (G4S)_1_ because the long linker did not work (**Supplementary Figure 2A**). The Bic05 long-linker version did not bind to TLR2-expressing cells and had no effect on moDCs (data not shown). Bic05 binding to donor moDCs was dose-dependent (**Supplementary Figure 5B**) and could be inhibited by anti-TLR2 or anti-DCIR scFvs (**Figure 2A**). The *K*_D_ of Bic05 on interferometry was 0.55 nM, comparable to that of the anti-TLR2 scFv (0.30 nM) (data not shown). Its antigenic target (DCIR) is expressed at a similar level to CD209 (**Supplementary Figure 3B**). Like Bic03, Bic05 induced a semi-mature phenotype of moDCs (**Figure 2B**; **Supplementary Figure 6B**) and a dose-dependent secretion of IL-10 and TGF-β1 (**Figures 2D–F**). However, Bic05 induced a lower level of PD-L1 than with zym (**Figure 2C**), a higher level of IFN-γ (**Figure 2F**), and a higher percentage of cells expressing CCR7 (**Supplementary Figure 7B**). Of note, in sharp contrast with zym, Bic05 increased the proportion of IL-10Rα– and IL-10Rα/IL-10Rβ−expressing cells (**Supplementary Figures 8A, B**). Like Bic03, Bic05 treatment of PBMCs dose-dependently induced IL-10 secretion (**Figures 2G, H**). Bic05 induced the secretion of TGF-β1, IL-6, IFN-γ, IL-12p70, and TNF-α at low levels; these cytokines were generally produced in lower quantities than with zym. By contrast, Bic05 did not induce IL-1β or IL-4 secretion, contrary to zym (**Figure 2H**).

### 3.5 Bic05-treated moDCs or PBMCs induce functional regulatory populations

**Figure 3:**
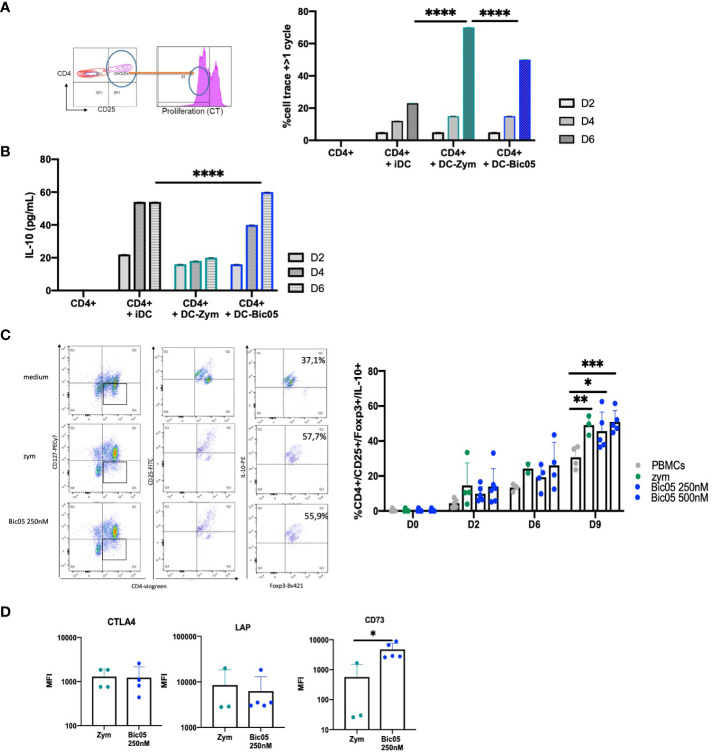
Bic05-induced regulatory populations in T-cell cultures. **(A)** Dot plot at day 2 and proportion of proliferative CD4^+^ T cells in MLR with moDCs treated with zym or Bic05 (250 nM) on days 2, 4, and 6 (one out of four experiments, ^****^p < 0.0001). **(B)** IL-10 measurement in MLR supernatant (*n* = 4, ^****^p < 0.0001). **(C)** Dot plots on day 9 of one donor in five and proportion of CD4^+^CD25^+^CD127^low^ Foxp3^+^IL-10^+^ T cells in PBMC cultures on days 0, 2, 6, and 9. IL-2 at 100 UI/mL was added on day 5 (*n* = 5, ^*^p < 0.005, ^**^p < 0.001, ^***^p < 0.0005). **(D)** MFI of CTLA-4, Lap, and CD73 expression on Treg populations (*n* = 5, ^*^p < 0.01). MLR, mixed lymphocyte reaction; MFI, mean fluorescence intensity.

Having phenotypically observed the differentiation of Treg populations with Bic03-treated DCs, a phenomenon that suggests the induction of tolDCs (28), we analyzed in further detail the properties of Bic05-pretreated DCs in coculture experiments. MLR with Bic05-pretreated moDCs and allogeneic CD4^+^/CD25^−^ cells led to an intermediate level of CD4^+^ T-cell proliferation as compared with inflammatory DCs and zym-pretreated DCs on day 6 (**Figure 3A**) as for iDCs and contrary to zym-pretreated DCs. Under the same conditions, Bic05-pretreated DCs induced IL-10 production in supernatants (**Figure 3B**).

**Figure 4:**
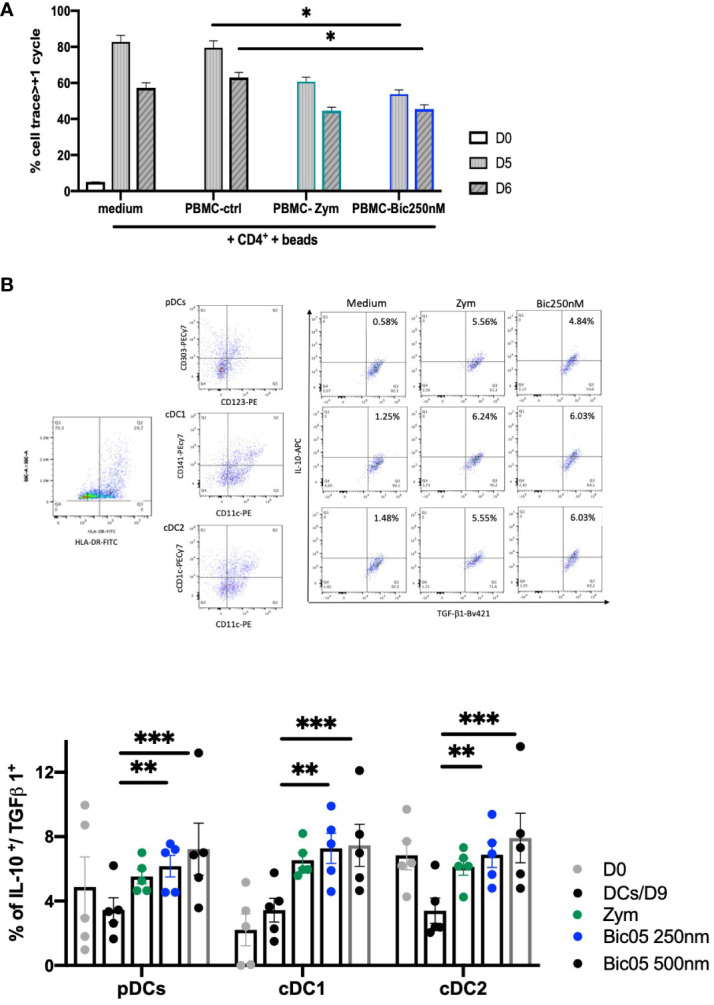
Bic05-induced regulatory populations in PBMC cultures. **(A)** Proliferation of CD4^+^+bead coculture with day-9 PBMCs treated with zym or Bic05 on days 0, 5, and 6 (^*^p < 0.01, one of three). **(B)** Dot plots (HLA-DR^+^, pDC, cDC1, cDC2, DCs IL-10^+^/TGF-β1^+^) at day 9 and percentage of IL-10^+^/TGF-β1^+^ DCs in total cell population on days 0 and 9 in PBMC cultures (*n* = 5, ^**^p < 0.0016, ^***^p < 0.0005).

In order to confirm the orientation toward Bic05-induced tolerance, we performed long-term cultures of PBMC with Bic05 concentrations of 250 nM and 500 nM at the maximal plateau of IL-10 secretion for 2, 6, and 9 days (**Figure 3C**). These high concentrations were chosen to anticipate Bic05 degradation during the long-term cultures. So, we demonstrated that Bic05 was able to increase the proportion of CD4^+^/CD127^low^/CD25^+^/Foxp3^+^/IL-10^+^ Tregs similar to with zym. Bic05 induced Treg-expressed LAP (membrane TGF-β1) and CTLA-4 to the same extent as that induced with zym but significantly greater CD73 induction (**Figure 3D**). Cells from Bic05-treated PBMC cultures on day 9 strongly inhibited the proliferation of allogeneic CD4^+/^CD25^−^ T cells induced by CD3^+^/CD28^+^ beads, as did zym-treated PBMCs (**Figure 4A**), and as expected from a mixture of tolerant DCs and functional Tregs (29).

In addition to the Treg compartment, we evaluated the ability of blood DCs to be modulated in long-term Bic05-treated PBMC cultures. Hence, pDCs were identified by CD123^+^/CD303^+^, cDC1 cells by CD11c^+^/CD141^+^, and cDC2 cells by CD11c^+^/CD1c^+^ labeling in the HLA-DR gate (30, 31). All circulating DC subsets, identified by their phenotypic profiles, secreted IL-10 and TGF-β1. The survival rate on day 9 of pDCs and cDC1^+^(cDC2) and DC CD141^+^(cDC1) number was increased immediately (**Figure 4B**) (32).

### 3.6 Evaluation of both bsAbs on SF cells from gout or RA patients

**Figure 5:**
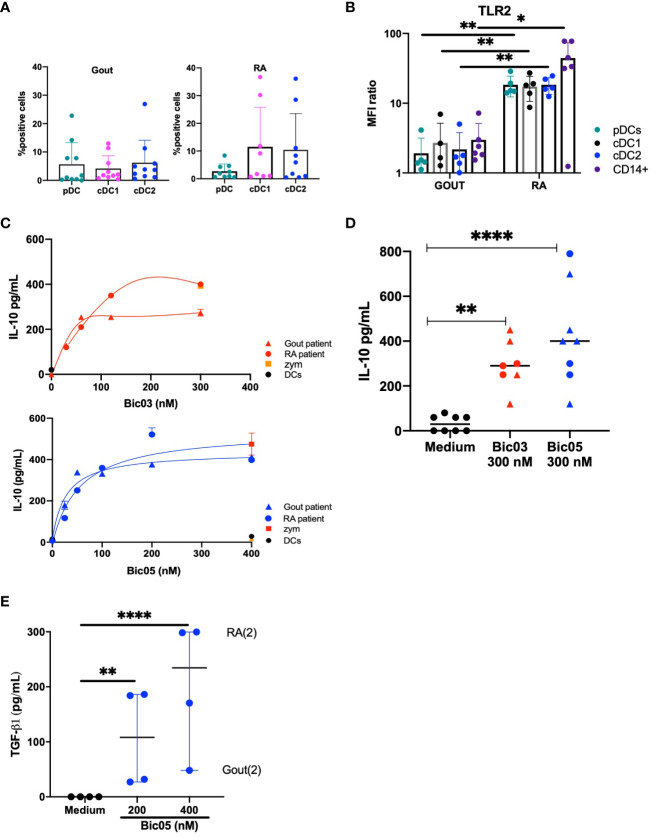
Both Bic-induced IL-10 and TGF-β1 secretion by SF cells. **(A)** Percentage of pDCs, cDC1, and cDC2 in total cells in SF from gout and RA patients (average % from *n* = 8 and 9 gout and RA patients, respectively). **(B)** MFI ratio of TLR2 expression of pDC, cDC1, cDC2, and CD14^+^ cells in SF from gout and RA patients (*n* = 6, ^*^p < 0.01, ^**^p < 0.001). **(C)** ELISA of IL-10 secretion in SF cell culture for 48 h for two of 19 patients, for Bic03 and Bic05 with RA (round) and gout (triangle) patients. **(D)** IL-10 secretion evaluated by ELISA in SF cell culture from both RA (round) and gout (triangle) patients with 300 nM Bic03 or Bic05 for 48 h (*n* = 7 [Bic03], *n* = 8 [Bic05]), ^**^p < 0.006, ^****^p < 0.0001). **(E)** TGF-β1 in SF cell culture with Bic05 (*n* = 4, ^**^p < 0.0016, ^****^p < 0.0001). SF, synovial fluid; RA, rheumatoid arthritis; MFI, mean fluorescence intensity.

To directly assess inflammatory DCs, we obtained SF cells from patients in a small cohort (NCT03416543) (**Supplementary Table S1**) of gout and RA patients, representing two kinds of inflammatory conditions. In accordance with what was previously reported (33), all three DC subsets were equally distributed in gout and RA SF (**Figure 5A**). TLR2 was significantly overexpressed in all three DC subsets and monocytes of RA as compared with gout monocytes and DCs (**Figure 5B**). TLR2 was coexpressed with CD209 in all DC subsets and with DCIR in all DC subsets in both diseases (**Supplementary Figures 9A–C**). By contrast, except for IL-12p70 and IFN-γ, the levels of inflammatory cytokines in SF did not differ between gout and RA disease (**Supplementary Figure 10**). SF from RA patients induced a higher secretion of IFN-γ and a lower secretion of IL-12p70 than that from gout patients.

After 48 h of incubation of purified SF cells, both Bics induced a similar dose-dependent increase in IL-10 secretion from SF from both diseases, reaching a plateau above 200 nM (**Figures 5C, D**). SF cells from both diseases showed significant TGF-β1 secretion under the same conditions (**Figure 5E**).

## 4 Discussion

In the present study, we sought to establish a new immune-therapeutic strategy using bsAbs targeting DCs to render them tolerant. This strategy tried to mimic what pathogens already do, forcing cooperation between CLRs and TLRs (34, 35). We envisioned inducing tolerogenic DCs by using bsAb fragments (Bics) against CLRs such as CD209 and DCIR and against TLR2 *in vitro* as a proof of concept.

To avoid uncontrolled DC modulation, our bsAb constructs met quality specifications with an in-depth analysis of the structure, solubility, and endotoxin contamination. The mass spectra showed little additive glycosylation only for Bic03 without changing the bsAb-binding properties, and purification with size-exclusion chromatography showed mostly all bsAbs in the monomeric form without aggregates. The biological effects observed require the interaction of both Bics with the DC surface. Both Bics exhibited a saturation binding curve on the moDC surface at 37°C, which suggests little or no endocytosis, contrary to what has been reported for monospecific Abs (26, 36). This observation clearly indicates that the forced tethering of the two antigens modifies their individual trafficking and functionality. The moDCs express high amounts of CD209 and DCIR on their surface and far less TLR2, which could explain why anti-TLR2 scFvs were more efficient in competing with Bics but also that the Bics were able to retain TLR2 at the cell surface and disturb its intracellular trafficking (37). Because none of the scFvs alone were able to induce IL-10 secretion by moDCs, the bridging may be necessary for bsAb properties.

The linker between both scFvs was also an important parameter of the bsAb functionality. We produced both short- and long-linker molecules for both Bics. For Bic05, short- and long-linker formats are bound on moDCs with a different curve progression. However, the Bic05 long linker did not induce moDC maturation or IL-10 secretion. We also did not observe TLR2 binding with the Bic05 long linker on HEK-TLR2 cells as compared with the short linker format (i.e., Bic05) (data not shown). G4S is usually considered a rigid link, but other studies in the lab showed that this type of linker could be flexible with real degrees of freedom (38). The long linker providing larger degrees of freedom to scFVs might be deleterious. The size of the long linker could allow for a U-shaped folding, which could block the TLR2 binding, which cannot occur with the short linker. This situation may also be due to the nature of the anti-DCIR scFv because this blockage was not observed with the Bic03 long linker. Nevertheless, it reinforces that binding to both CLR and TLR is mandatory to induce IL-10 secretion from moDCs. Both bsAbs could behave in the same way. In Dillon’s article, *cis* activation has been proposed for a zymosan mechanism with a bridge between TLR2 and dectin-1 and based on the transduction signal request to obtain both DC IL-10 and TGF-β1 secretion (17). Moreover, the differences in the transduction signal observed between simple TLR2 agonists such as Pam-3cys and zymosan comfort the *cis* activation hypotheses. As we obtained both tolerant cytokine secretions, we can hypothesize that *cis* activation is playing in our case, for the same signal transduction reasons.

The required specification for tolDCs, despite no definitive profile, is their capacity to induce or activate Tregs associated with a semimature profile and the expression of inhibitory molecules such as PD-L1 or ILT3 (39). Our Bic-treated DCs induced allogenic Tregs mainly via IL-10 and TGF-β1 secretion, as previously reported (40). Additionally, the expression of several tolerance markers on both Bic-treated DCs, such as PD-L1, ILT3, or ILT4, reinforces the hypothesis of their capacity to drive Treg differentiation (41). Both Bics induced CCR7 expression on moDCs, as in other tolDC models (42). The immune tolerance induced by both Bics seems different from that induced by zym. Indeed, zym-induced proinflammatory cytokines such as IL-12p70 and TNF-α, together with anti-inflammatory cytokines. However, both Bics induced fewer inflammatory cytokines than zym and no TNF-α. The differences from zym treatment can be explained by the different CLR cross-linkings to TLR2: dectin-1 for zym and CD209 and DCIR for Bics. Moreover, Bic05 induced a higher number of IL-10Rα–expressing cells as compared with zym and dose-dependently, probably rendering them more sensitive to IL-10 via an autocrine loop (43). DC survival depends on complex transduction signals and CD40-dependent T-cell interaction (44, 45), but, usually, DCs die in PBMC cultures for more than 5 days. DCs are persistent and long-lived in the tumor environment, associated with glucocorticoid-induced leucine zipper (GILZ) gene expression (46). So, the up to 9-day survival of the three DC subsets in Bic05-treated PBMC cultures might aid their protolerogenic orientation (45, 47). Finally, both Bics seemed able to induce tolDCs, which are able to migrate to lymph nodes, secreting mainly IL-10 and TGF-β1 and inducing functional Treg differentiation.

For Bic03, we identified CD4^+^/CD25^+^ cells that were Foxp3^+^ or IL-10^+^, which questions the real nature of these induced Tregs. The heterogeneity of Tregs between thymus Tregs and peripheral Tregs is acknowledged (48). The Foxp3 molecule remains the main marker of natural Tregs in the thymus, as do several other Treg phenotypes, such as Tr1, which solely secrete IL-10 but do not express Foxp3. At least two Treg populations that are not mutually exclusive may have been induced by culture. We observed a discrepancy in the proportion of these populations. The iDCs induced more Foxp3^+^ than IL-10^+^ cells, in line with the literature (49). Zym induced the same proportion of both populations, at least in mice (17). For Bic03, the lowest concentration induced a comparable profile with iDCs, whereas the highest concentrations did not favor Foxp3^+^ cell differentiation. Because Bic03 modulates moDC phenotypes dose-dependently for all studied markers, a high expression of inhibitory markers may not necessarily lead to Foxp3^+^ cell differentiation, but the few differentiated cells were able to trigger IL-10 secretion of all CD4^+^ cells in culture, in line with the “infectious tolerance” concept (50). A high molecular concentration of Bic seems unnecessary and even counterproductive, as we also observed for Bic05 and Treg differentiation (**Figure 3C**). For Bic05, induced Tregs were both IL-10^+^ and Foxp3^+^ and expressed more CD73 than zym-induced Tregs, which suggests the participation of purinergic signals (51, 52). Finally, both Bics seemed able to confer regulatory properties to PBMC populations with the development of particular Tregs.

We further studied SF cells from gout and RA patients to evaluate whether both Bics could reverse inflammation. We found higher TLR2 expression in SF cells from RA than in gout patients (53). We cite the following evidence. First, low TLR2 expression in moDCs did not prevent IL-10 secretion (**Figures 1D**, **2D** and **5C**). Next, DCIR and TLR2 expression in PBMC populations seemed to mainly result in the CD14^+^ pool (**Supplementary Figure 7C**). More than DCs, other monocyte populations infiltrate joints in both RA and gout (54–56). So, these populations, which are highly represented in inflammatory diseases, could be the main target of Bics because they are more numerous than DCs and their phenotype is sensitive to inflammation (57). For Bics, any cell expressing the appropriate targets is acceptable; even the important role of DCs in RA has been noted in the self-antigen presentation, DC–T-cell cooperation, and inflammation (58, 59). Second, both Bics changed inflamed synovial cells from both RA and gout into IL-10-secreting cells, with a plateau observed above 200 nM. The amount of IL-10 secretion after 48 h peaked, whatever the secretion kinetics and number of involved cells. This peak could be due to the sum of IL-10 from targeted cells plus IL-10-sensitive cells that may also secrete IL-10. Moreover, there is no need to target a large number of cells to obtain a large amount of IL-10 secretion and induce a state of immune tolerance; this is the concept of infectious tolerance (60). Finally, both Bics might represent an alternative form of local or systemic therapy.

The other perspective is whether this concept of PRR bridging is applicable to other TLRs and other pairs of receivers. Our related work seems to show what occurred with two different models involving TLR2 and two CLRs. However, this concept could apply to other TLR–CLR pairs. In humans, 10 TLRs and 162 CLRs have been identified (61). This is a large number of pairs to check and opens up a vast field of investigation into models of molecular cooperation between receptors. In contrast, these cooperative TLR–CLR may not lead systematically to immune tolerance but also to inflammation, cell apoptosis, or other types of cell activation. The use of bsAbs in both therapy and basic immunology seems to have a promising future.

Here, we provide one recipe to train human DCs to become tolerant by using a new type of bsAb mimicking the tethering performed by pathogens (17). These Bics revealed a mode of control of inflammation by the cooperation of TLR2 with several CLRs (62). They may represent a new strategy for treating inflammatory diseases.

## Data availability statement

The original contributions presented in the study are included in the article/**Supplementary Material**. Further inquiries can be directed to the corresponding author.

## Ethics statement

The studies involving humans were approved by (ID RCB: 2017-A02678-45) and approved by the institutional review board – “Comité de Protection des Personnes - Ile de France VIII” (CPP: 17 11 76). The studies were conducted in accordance with the local legislation and institutional requirements. The participants provided their written informed consent to participate in this study.

## Author contributions

LL: Formal Analysis, Investigation, Methodology, Writing – original draft. MG: Investigation, Methodology, Writing – original draft. NDK-N: Investigation, Methodology, Writing – original draft. ZL: Investigation, Writing – original draft. DMe: Investigation, Writing – original draft. AP: Formal Analysis, Investigation, Methodology, Writing – original draft, Writing – review & editing. TL: Methodology, Writing – original draft. AdT: Investigation, Writing – original draft. JP: Methodology, Resources, Writing – original draft. DMu: Funding acquisition, Project administration, Writing – original draft, Writing – review & editing. GL: Investigation, Methodology, Writing – original draft. NA: Conceptualization, Formal Analysis, Investigation, Methodology, Writing – original draft, Writing – review & editing. HW: Formal Analysis, Funding acquisition, Project administration, Writing – original draft, Writing – review & editing. FV-R: Conceptualization, Formal Analysis, Funding acquisition, Investigation, Methodology, Project administration, Supervision, Writing – original draft, Writing – review & editing.

## References

[1] Kowalczyk A D’Souza CA Zhang L. Cell-extrinsic CTLA4-mediated regulation of dendritic cell maturation depends on STAT3: Molecular immunology. Eur J Immunol. (2014) 44:1143–55. doi: 10.1002/eji.201343601 24338929

[2] Garris CS Wong JL Ravetch JV Knorr DA. Dendritic cell targeting with Fc-enhanced CD40 antibody agonists induces durable antitumor immunity in humanized mouse models of bladder cancer. Sci Transl Med. (2021) 13:eabd1346. doi: 10.1126/scitranslmed.abd1346 34011627 PMC8325152

[3] Gauvreau GM Boulet L-P Cockcroft DW FitzGerald JM Mayers I Carlsten C. OX 40L blockade and allergen-induced airway responses in subjects with mild asthma. Clin Exp Allergy. (2014) 44:29–37. doi: 10.1111/cea.12235 24224471 PMC4253735

[4] Muik A Adams HC Gieseke F Altintas I Schoedel KB Blum JM. DuoBody-CD40x4-1BB induces dendritic-cell maturation and enhances T-cell activation through conditional CD40 and 4-1BB agonist activity. J Immunother Cancer. (2022) 10:e004322. doi: 10.1136/jitc-2021-004322 35688554 PMC9189854

[5] Sung E Ko M Won J Jo Y Park E Kim H. LAG-3xPD-L1 bispecific antibody potentiates antitumor responses of T cells through dendritic cell activation. Mol Ther. (2022) 30:2800–16. doi: 10.1016/j.ymthe.2022.05.003 PMC9372323 35526096

[6] Steinman RM Hawiger D Nussenzweig MC. Tolerogenic dendritic cells. Annu Rev Immunol. (2003) 21:685–711. doi: 10.1146/annurev.immunol.21.120601.141040 12615891

[7] Balan S Saxena M Bhardwaj N. Dendritic cell subsets and locations. Int Rev Cell Mol Biol. (2019) 348:1–68. doi: 10.1016/bs.ircmb.2019.07.004 31810551

[8] Collin M Ginhoux F. Human dendritic cells. Semin Cell Dev Biol. (2019) 86:1–2. doi: 10.1016/j.semcdb.2018.04.015 29727728

[9] Segura E Amigorena S. Inflammatory dendritic cells in mice and humans. Trends Immunol. (2013) 34:440–5. doi: 10.1016/j.it.2013.06.001 23831267

[10] Lamendour L Deluce-Kakwata-Nkor N Mouline C Gouilleux-Gruart V Velge-Roussel F. Tethering innate surface receptors on dendritic cells: A new avenue for immune tolerance induction? Int J Mol Sci. (2020) 21:5259. doi: 10.3390/ijms21155259 32722168 PMC7432195

[11] Steinman RM. The control of immunity and tolerance by dendritic cell. Pathol Biol (Paris). (2003) 51:59–60. doi: 10.1016/S0369-8114(03)00096-8 12801800

[12] Sato K Uto T Fukaya T Takagi H. Regulatory dendritic cells. Curr Top Microbiol Immunol. (2017) 410:47–71. doi: 10.1007/82_2017_60 28900681

[13] Liu Y Chen Y Liu FQ Lamb JR Tam PKH. Combined treatment with triptolide and rapamycin prolongs graft survival in a mouse model of cardiac transplantation. Transplant Int. (2008) 21:483–94. doi: 10.1111/j.1432-2277.2007.00630.x 18266776

[14] Yang J Liu L Yang Y Kong N Jiang X Sun J. Adoptive cell therapy of induced regulatory T cells expanded by tolerogenic dendritic cells on murine autoimmune arthritis. J Immunol Res. (2017) 2017:1–13. doi: 10.1155/2017/7573154 PMC5494067 28702462

[15] Feng D Wang Y Liu Y Wu L Li X Chen Y. DC-SIGN reacts with TLR-4 and regulates inflammatory cytokine expression via NF-κB activation in renal tubular epithelial cells during acute renal injury: DC-SIGN/TLR-4 regulates NF-κB activation. Clin Exp Immunol. (2018) 191:107–15. doi: 10.1111/cei.13048 PMC5721233 28898406

[16] Seno A Maruhashi T Kaifu T Yabe R Fujikado N Ma G. Exacerbation of experimental autoimmune encephalomyelitis in mice deficient for DCIR, an inhibitory C-type lectin receptor. Exp Anim. (2015) 64:109–19. doi: 10.1538/expanim.14-0079 PMC4427725 26176030

[17] Dillon S Agrawal S Banerjee K Letterio J Denning TL Oswald-Richter K. Yeast zymosan, a stimulus for TLR2 and dectin-1, induces regulatory antigen-presenting cells and immunological tolerance. J Clin Invest. (2006) 116:916–28. doi: 10.1172/JCI27203 PMC1401484 16543948

[18] Koymans KJ Goldmann O Karlsson CAQ Sital W Thänert R Bisschop A. The TLR2 antagonist staphylococcal superantigen-like protein 3 acts as a virulence factor to promote bacterial pathogenicity *in vivo* . J Innate Immun. (2017) 9:561–73. doi: 10.1159/000479100 28858870

[19] Hajishengallis G Lamont RJ. Breaking bad: Manipulation of the host response by Porphyromonas gingivalis. Eur J Immunol. (2014) 44:328–38. doi: 10.1002/eji.201344202 PMC3925422 24338806

[20] Ciaston I Dobosz E Potempa J Koziel J. The subversion of toll-like receptor signaling by bacterial and viral proteases during the development of infectious diseases. Mol Aspects Med. (2022) 88:101143. doi: 10.1016/j.mam.2022.101143 36152458 PMC9924004

[21] Dawod B Haidl ID Azad MB Marshall JS. Toll-like receptor 2 impacts the development of oral tolerance in mouse pups via a milk-dependent mechanism. J Allergy Clin Immunol. (2020) 146:631–641.e8. doi: 10.1016/j.jaci.2020.01.049 32068020

[22] Kirschning CJ Dreher S Maaß B Fichte S SChade J Köster M. Generation of anti-TLR2 intrabody mediating inhibition of macrophage surface TLR2 expression and TLR2-driven cell activation. BMC Biotechnol. (2010) 10:31. doi: 10.1186/1472-6750-10-31 20388199 PMC2873280

[23] Lakhrif Z Pugnière M Henriquet C di Tommaso A Dimier-Poisson I Billiald P. A method to confer Protein L binding ability to any antibody fragment. mAbs. (2016) 8:379–88. doi: 10.1080/19420862.2015.1116657 PMC4966575 26683650

[24] Bourquard T Musnier A Puard V Tahir S Ayoub MA Jullian Y. MAbTope: A method for improved epitope mapping. J Immunol. (2018) 201:3096–105. doi: 10.4049/jimmunol.1701722 30322966

[25] Webb B Sali A. Comparative protein structure modeling using MODELLER. Curr Protoc Bioinf. (2016) 54:5.6. doi: 10.1002/cpbi.3 PMC5031415 27322406

[26] Tacken PJ Ginter W Berod L Cruz LJ Joosten B Sparwasser T. Targeting DC-SIGN via its neck region leads to prolonged antigen residence in early endosomes, delayed lysosomal degradation, and cross-presentation. Blood. (2011) 118:4111–9. doi: 10.1182/blood-2011-04-346957 21860028

[27] Lutz MB. Therapeutic potential of semi-mature dendritic cells for tolerance induction. Front Immun. (2012) 3:123. doi: 10.3389/fimmu.2012.00123 PMC3355325 22629255

[28] Dáňová K Grohová A Strnadová P Funda DP Šumník Z Lebl J. Tolerogenic dendritic cells from poorly compensated type 1 diabetes patients have decreased ability to induce stable antigen-specific T cell hyporesponsiveness and generation of suppressive regulatory T cells. JI. (2017) 198:729–40. doi: 10.4049/jimmunol.1600676 27927966

[29] Grover P Goel PN Greene MI. Regulatory T cells: regulation of identity and function. Front Immunol. (2021) 12:750542. doi: 10.3389/fimmu.2021.750542 34675933 PMC8524049

[30] Collin M Bigley V. Human dendritic cell subsets: an update. Immunology. (2018) 154:3–20. doi: 10.1111/imm.12888 29313948 PMC5904714

[31] Abbas A Vu Manh T-P Valente M Collinet N Attaf N Dong C. The activation trajectory of plasmacytoid dendritic cells *in vivo* during a viral infection. Nat Immunol. (2020) 21:983–97. doi: 10.1038/s41590-020-0731-4 PMC7610367 32690951

[32] McDaniel MM Kottyan LC Singh H Pasare C. Suppression of inflammasome activation by IRF8 and IRF4 in cDCs is critical for T cell priming. Cell Rep. (2020) 31:107604. doi: 10.1016/j.celrep.2020.107604 32375053 PMC7325595

[33] Cavanagh LL Boyce A Smith L Padmanabha J Filgueira L Pietschmann P. Rheumatoid arthritis synovium contains plasmacytoid dendritic cells. Arthritis Res Ther. (2005) 7:R230. doi: 10.1186/ar1467 15743469 PMC1065313

[34] Oliveira-Nascimento L Massari P Wetzler LM. The role of TLR2 in infection and immunity. Front Immunol. (2012) 3:79. doi: 10.3389/fimmu.2012.00079 22566960 PMC3342043

[35] Kawai T Akira S. Toll-like receptors and their crosstalk with other innate receptors in infection and immunity. Immunity. (2011) 34:637–50. doi: 10.1016/j.immuni.2011.05.006 21616434

[36] Meyer-Wentrup F Benitez-Ribas D Tacken PJ Punt CJA Figdor CG de Vries IJM. Targeting DCIR on human plasmacytoid dendritic cells results in antigen presentation and inhibits IFN- production. Blood. (2008) 111:4245–53. doi: 10.1182/blood-2007-03-081398 18258799

[37] Musilova J Mulcahy ME Kuijk MM McLoughlin RM Bowie AG. Toll-like receptor 2–dependent endosomal signaling by Staphylococcus aureus in monocytes induces type I interferon and promotes intracellular survival. J Biol Chem. (2019) 294:17031–42. doi: 10.1074/jbc.RA119.009302 PMC6851302 31558608

[38] Aubrey N Gouilleux-Gruart V Dhommée C Mariot J Boursin F Albrecht N. Anticalin N- or C-terminal on a monoclonal antibody affects both production and *in vitro* functionality. Antibodies. (2022) 18:1–18. doi: 10.3390/antib11030054 PMC9397084 35997348

[39] Iberg CA Hawiger D. Natural and induced tolerogenic dendritic cells. J Immunol. (2020) 204:733–44. doi: 10.4049/jimmunol.1901121 PMC7006631 32015076

[40] Boks MA Kager-Groenland JR Haasjes MSP Zwaginga JJ van Ham SM ten Brinke A. IL-10-generated tolerogenic dendritic cells are optimal for functional regulatory T cell induction — A comparative study of human clinical-applicable DC. Clin Immunol. (2012) 142:332–42. doi: 10.1016/j.clim.2011.11.011 22225835

[41] Arboleda JF García LF Alvarez CM. [ILT3+/ILT4+ tolerogenic dendritic cells and their influence on allograft survival]. Biomedica. (2011) 31:281–95. doi: 10.1590/S0120-41572011000200017 22159546

[42] Lagaraine C Hoarau C Chabot V Velge-roussel F Lebranchu Y. Human mycophenolic acid treated dendritic cells have immature co-stimulary abilities but mature migratory phenotype. J Leukocyte Biol. (2005) 17:351–63. doi: 10.1093/intimm/dxh215 15710908

[43] Ye H Pan J Cai X Yin Z Li L Gong E. IL−10/IL−10 receptor 1 pathway promotes the viability and collagen synthesis of pulmonary fibroblasts originated from interstitial pneumonia tissues. Exp Ther Med. (2022) 24:518. doi: 10.3892/etm.2022.11445 35837039 PMC9257754

[44] Maney NJ Reynolds G Krippner-Heidenreich A Hilkens CMU. Dendritic cell maturation and survival are differentially regulated by TNFR1 and TNFR2. JI. (2014) 193:4914–23. doi: 10.4049/jimmunol.1302929 PMC4896387 25288570

[45] Miga AJ Masters SR Durell BG Gonzalez M Jenkins MK Maliszewski C. Dendritic cell longevity and T cell persistence is controlled by CD154-CD40 interactions. Eur J Immunol. (2001) 31:959–65. doi: 10.1002/1521-4141(200103)31:3<959::AID-IMMU959>3.0.CO;2-A 11241301

[46] Lebson L Wang T Jiang Q Whartenby KA. Induction of the glucocorticoid-induced leucine zipper gene limits the efficacy of dendritic cell vaccines. Cancer Gene Ther. (2011) 18:563–70. doi: 10.1038/cgt.2011.23 PMC3138804 21546924

[47] Bourque J Hawiger D. Life and death of tolerogenic dendritic cells. Trends Immunol. (2023) 44(2):110–8. doi: 10.1016/j.it.2022.12.006 PMC9892261 36599743

[48] Savage PA Klawon DEJ Miller CH. Regulatory T cell development. Annu Rev Immunol. (2020) 38:421–53. doi: 10.1146/annurev-immunol-100219-020937 31990619

[49] Jonuleit H Schmitt E Stassen M Tuettenberg A Knop J Enk AH. Identification and functional characterization of human CD4(+)CD25(+) T cells with regulatory properties isolated from peripheral blood. J Exp Med. (2001) 193:1285–94. doi: 10.1084/jem.193.11.1285 PMC2193380 11390435

[50] Kendal AR Waldmann H. Infectious tolerance: therapeutic potential. Curr Opin Immunol. (2010) 22:560–5. doi: 10.1016/j.coi.2010.08.002 20829013

[51] Alam MS Kurtz CC Rowlett RM Reuter BK Wiznerowicz E Das S. CD73 is expressed by human regulatory T helper cells and suppresses proinflammatory cytokine production and *Helicobacter felis* –induced gastritis in mice. J Infect Dis. (2009) 199:494–504. doi: 10.1086/596205 19281303 PMC3047419

[52] Da M Chen L Enk A Ring S Mahnke K. The multifaceted actions of CD73 during development and suppressive actions of regulatory T cells. Front Immunol. (2022) 13:914799. doi: 10.3389/fimmu.2022.914799 35711418 PMC9197450

[53] Coutant F Pin J-J Miossec P. Extensive phenotype of human inflammatory monocyte-derived dendritic cells. Cells. (2021) 10:1663. doi: 10.3390/cells10071663 34359833 PMC8307578

[54] Cren M Nziza N Carbasse A Mahe P Dufourcq-Lopez E Delpont M. Differential accumulation and activation of monocyte and dendritic cell subsets in inflamed synovial fluid discriminates between juvenile idiopathic arthritis and septic arthritis. Front Immunol. (2020) 11:1716. doi: 10.3389/fimmu.2020.01716 32849606 PMC7411147

[55] Theeuwes WF Di Ceglie I Dorst DN Blom AB Bos DL Vogl T. CD64 as novel molecular imaging marker for the characterization of synovitis in rheumatoid arthritis. Arthritis Res Ther. (2023) 25:158. doi: 10.1186/s13075-023-03147-y 37653557 PMC10468866

[56] Liu L Zhu L Liu M Zhao L Yu Y Xue Y. Recent insights into the role of macrophages in acute gout. Front Immunol. (2022) 13:955806. doi: 10.3389/fimmu.2022.955806 35874765 PMC9304769

[57] Coutant F. Shaping of monocyte-derived dendritic cell development and function by environmental factors in rheumatoid arthritis. IJMS. (2021) 22:13670. doi: 10.3390/ijms222413670 34948462 PMC8708154

[58] Wehr P Purvis H Law S-C Thomas R. Dendritic cells, T cells and their interaction in rheumatoid arthritis. Clin Exp Immunol. (2019) 196:12–27. doi: 10.1111/cei.13256 30589082 PMC6422662

[59] Meley D Héraud A Gouilleux-Gruart V Ivanes F Velge-Roussel F. Tocilizumab contributes to the inflammatory status of mature dendritic cells through interleukin-6 receptor subunits modulation. Front Immunol. (2017) 8:926. doi: 10.3389/fimmu.2017.00926 28861079 PMC5561017

[60] Waldmann H Graca L. Infectious tolerance. What are we missing? Cell Immunol. (2020) 354:104152. doi: 10.1016/j.cellimm.2020.104152 32585469

[61] Scur M Parsons BD Dey S Makrigiannis AP. The diverse roles of C-type lectin-like receptors in immunity. Front Immunol. (2023) 14:1126043. doi: 10.3389/fimmu.2023.1126043 36923398 PMC10008955

[62] Vogelpoel LTC Hansen IS Visser MW Nagelkerke SQ Kuijpers TW Kapsenberg ML. FcγRIIa cross-talk with TLRs, IL-1R, and IFNγR selectively modulates cytokine production in human myeloid cells. Immunobiology. (2015) 220:193–9. doi: 10.1016/j.imbio.2014.07.016 25108563

