# Supplementary material for: Bispecific antibodies tethering innate receptors induce human tolerant-dendritic cells and regulatory T cells
Source: Front Immunol. 2024 Mar 26;15:1369117. doi: 10.3389/fimmu.2024.1369117 (PMC11005913; doi:10.3389/fimmu.2024.1369117)
Supplement: Supplementary file 1 [file DataSheet_1.docx]

Bispecific antibodies tethering innate receptors induce human tolerant dendritic cells and regulatory T cells

Lamendour et al., 2024

Supplementary Material

## Supplementary Figures

## Supplementary Figure 1

## Supplementary Figure 2

## Supplementary Figure 3

## Supplementary Figure 4

## Supplementary Figure 5

## Supplementary Figure 6

## Supplementary Figure 7

## Supplementary Figure 8

## Supplementary Figure 9

## Supplementary Figure 10

**1.12 Supplementary Table 1.**


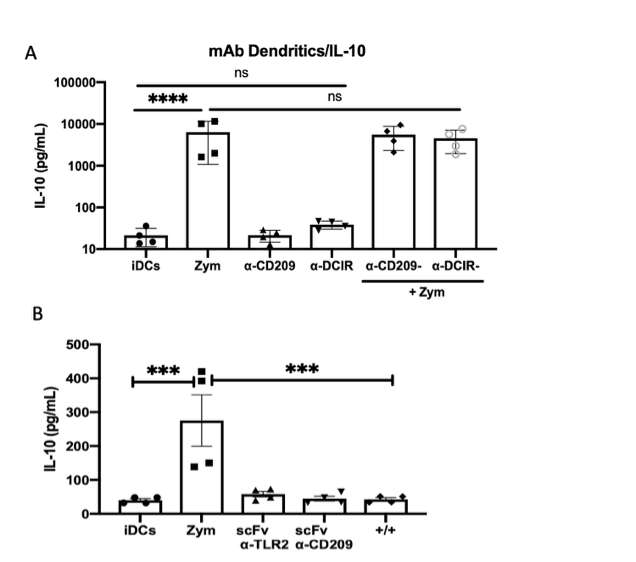


**Supplementary Figure 1. *IL-10 secretion of control monoclonal Antibodies (mAbs) and Single chain variable Fragment (scFvs).*** *(****A****) IL-10 secretion of moDCs treated with mAbs against CD209 or DCIR, in the presence of zymosan (zym, 5 μg/mL) (n=4, mean ± SD, ****p<0.0001)* *(****B****) IL-10 secretion of moDCs in the presence of zym (5 μg/mL), scFv anti-TLR2, anti-CD209 and both scFvs (n=4, mean ± SD, ***p<0.001).*

**
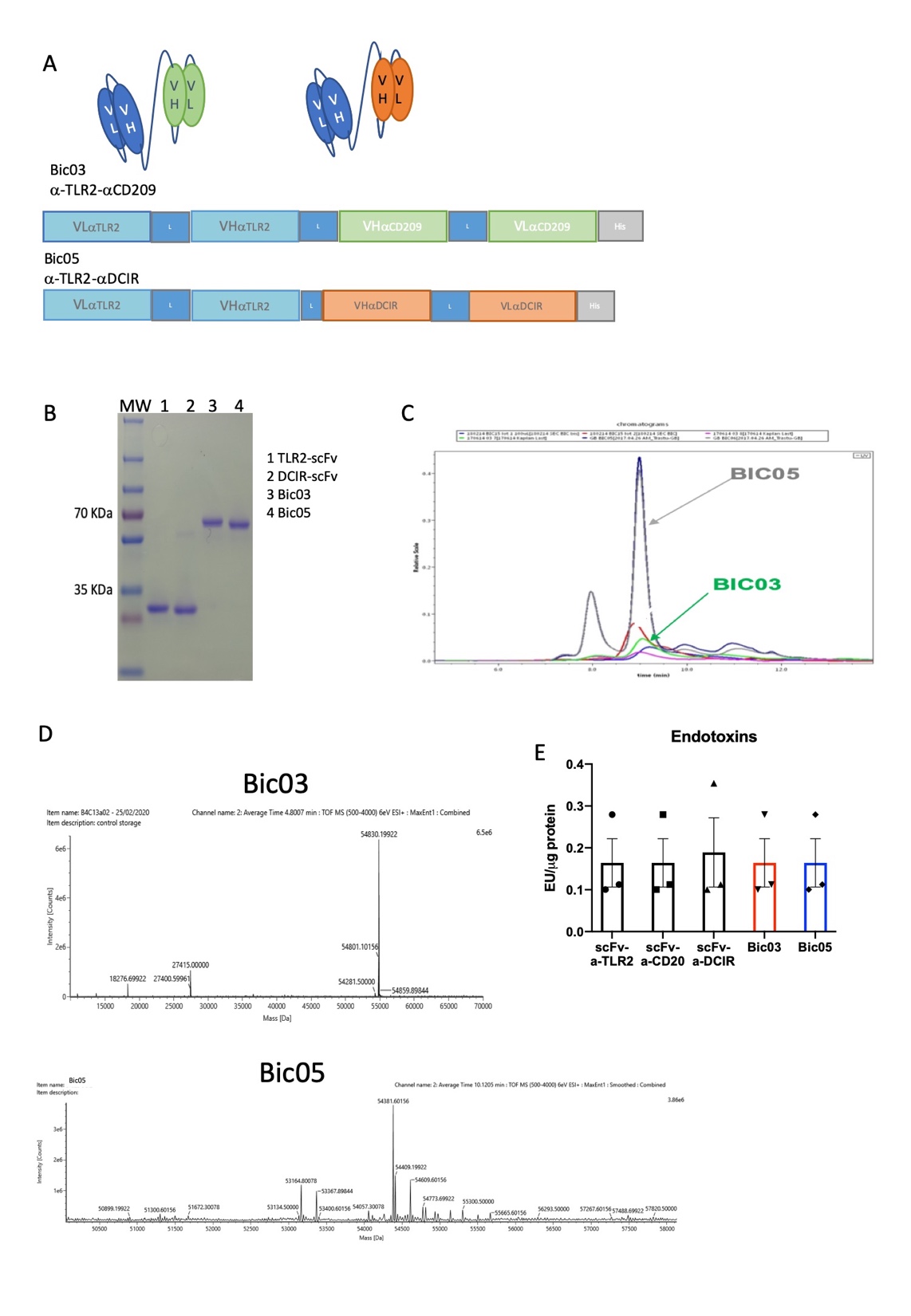
**

**Supplementary Figure 2. *Single chain variable Fragment (scFv) in Tandem αTLR2xαCLR (Bic03-Bic05)*** *(****A****) Schematic of Bic03 and Bic05: variable domain Heavy chain (VH)/variable domain light chain (VL) of anti-TLR2 mAb in light blue, anti-CD209 mAb in green, anti-DCIR in orange, G4S linkers in blue, histidine tag in gray. (****B****) SDS-PAGE of scFvs and Bic03, Bic05. (****C****) Size-exclusion chromatography of both Bics on a calibrated Superdex 200 Increase 10/300 GL column. (****D****) Mass spectra of Bic03 and Bic05. (****E****) Endotoxin measurements by LAL ELISA (n=3, mean ± SD).*


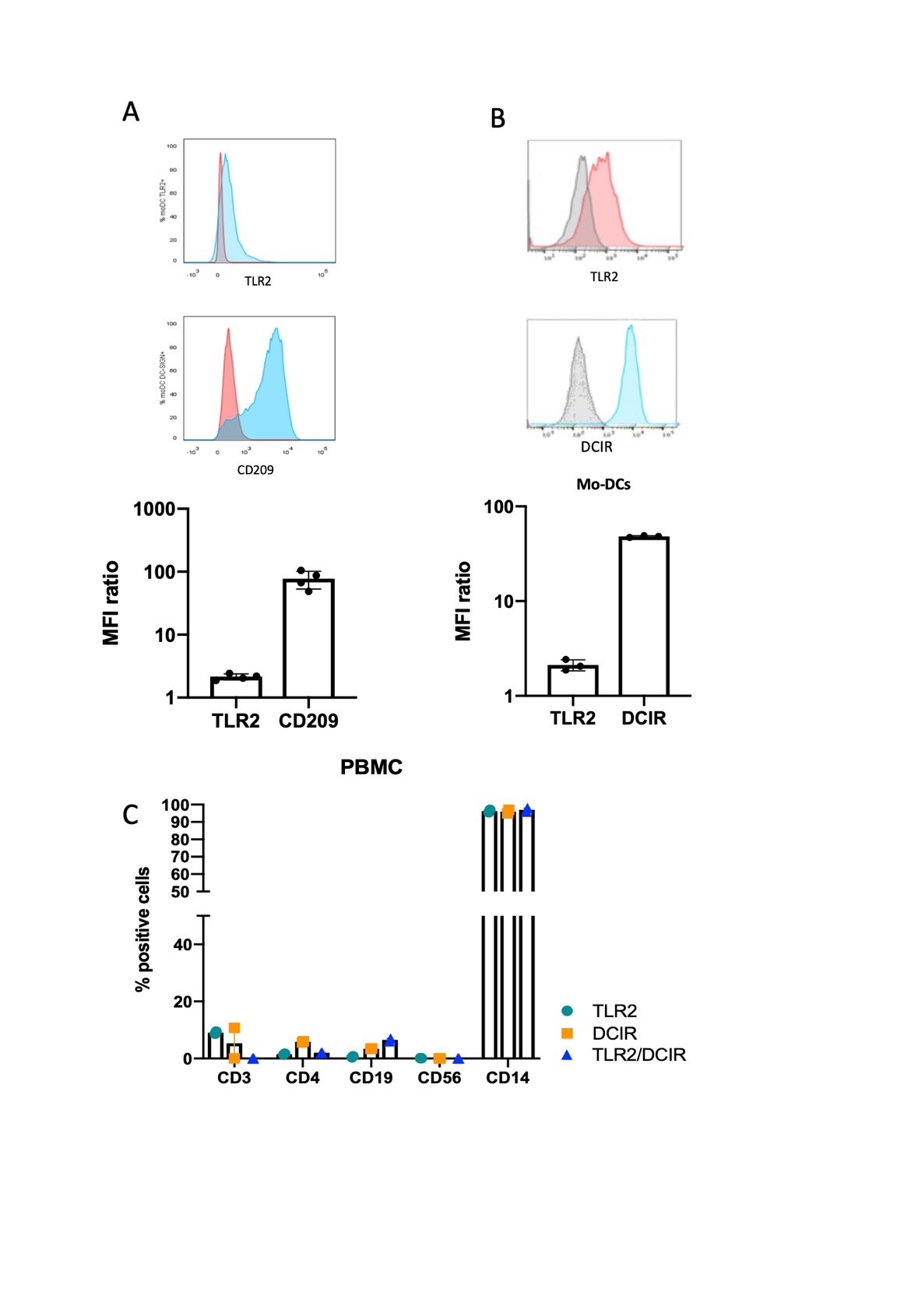


**Supplementary Figure 3**. ***Expression of target antigens on cells****. Flow cytometry of expression of (****A****) TLR2 and CD209 expressions on moDCs (n=7, mean ± SD), (****B****) TLR2 and DCIR expressions on moDCs (n= 7, mean ± SD), and (****C****) TLR2 and DCIR expressions in PBMC subsets (n=3, mean ± SD).*


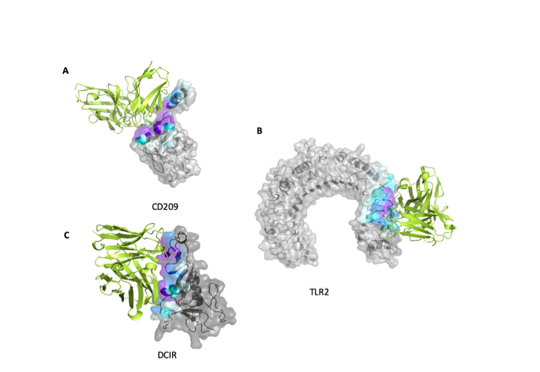


**Supplementary Figure 4.** **Epitope mapping**. *Predictive epitope mapping and Ab-target complex on (****A****) hCD209, (****C****) hDCIR and (****B)*** *hTLR2. Color codes: CD209 (aa 250 to 404), DCIR (aa 1-237) and TLR2 (aa 27-506) in grey. The predicted residues of the different molecules are colored according to their presence in the 20 best-ranked conformations, from violet (present in more than 20 of 30 conformations) to light cyan (present in 5 to 10 conformations). Amino acids in grey are not considered potential epitope residues.*


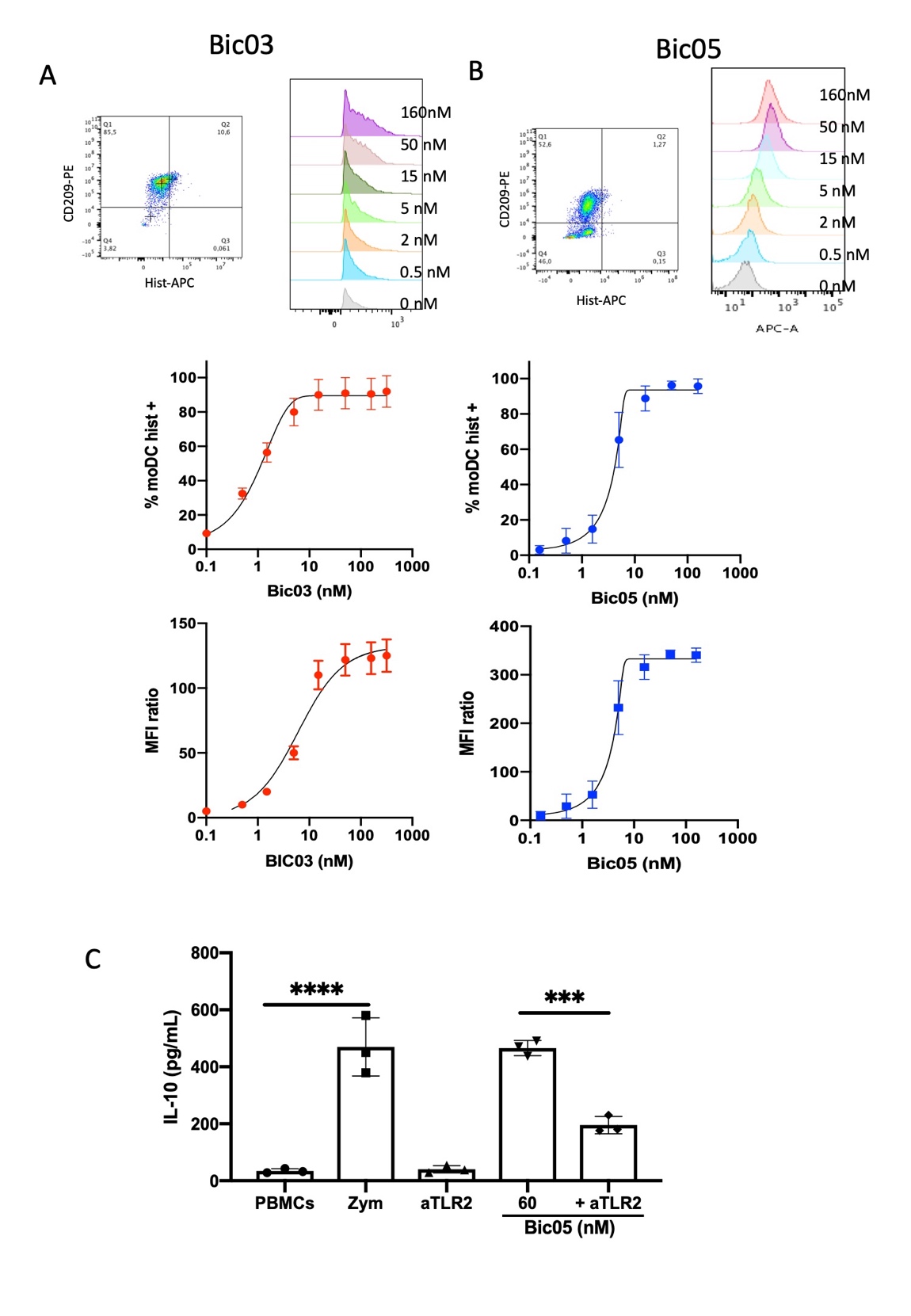


**Supplementary Figure 5.** **Binding properties of Bics.** *(****A****) Bic03 moDC 2-hr binding revealed by anti-His-APC on flow cytometry (1 of 5 experiments) expressed as % of positive cells or MFI ratio. (****B****) Bic05 moDC binding revealed by anti-His-APC on flow cytometry (1 of 7 experiments). (****C****) IL-10 secretion of PBMCs with Bic05 in the presence of anti-TLR2 mAb (3 μg/mL) (n=3, mean ± SD, ****p>0.0001, ***p<0.0004). MFI ratio, mean of fluorescent ratio.*


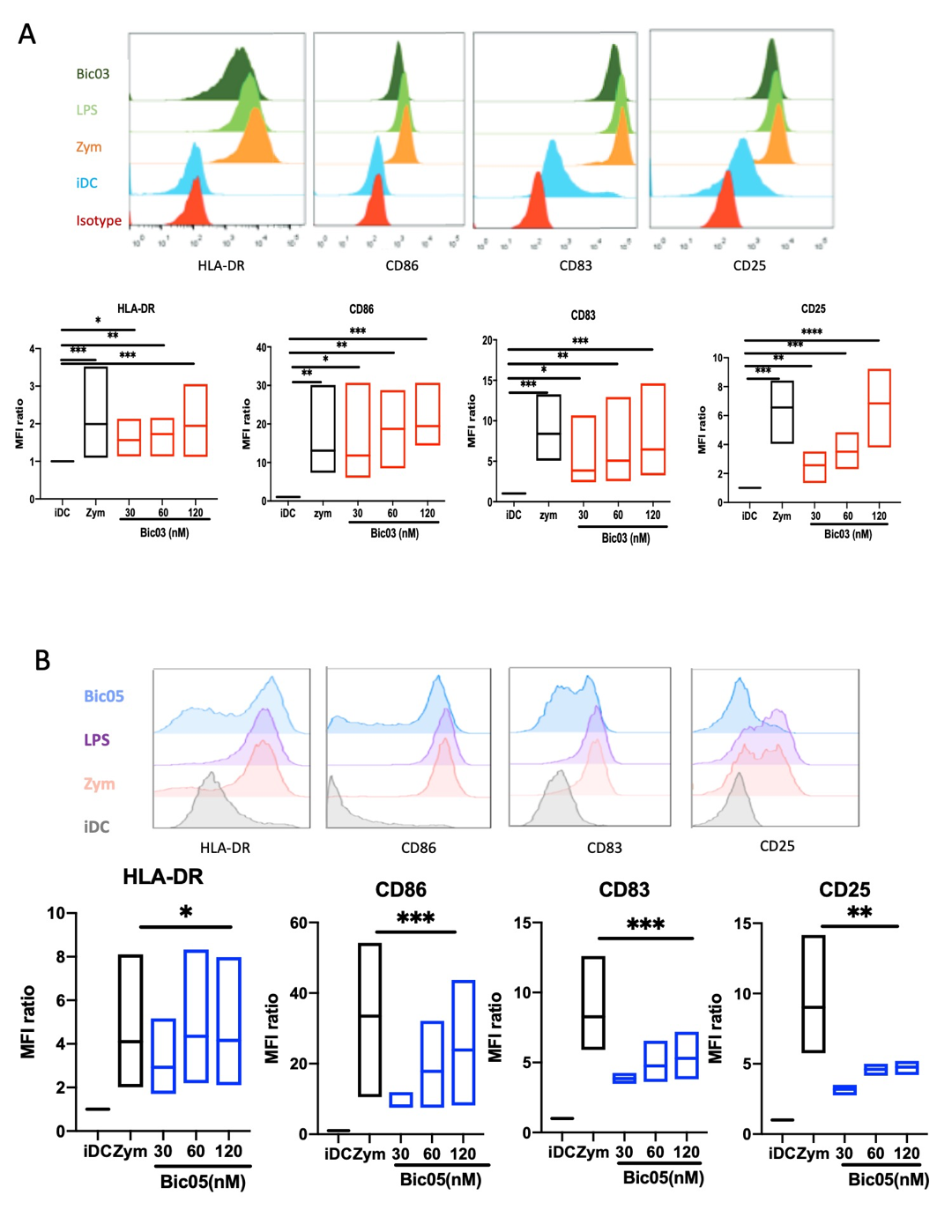


**Supplementary Figure 6.** *Maturation induced by (****A****) Bic03 (n=7, *p>0.01, **p<0.001) and (****B****) Bic05 (n=8, ***p<0.001, **p<0.004) revealed by HLA-DR, CD86, CD83, and CD25 expression on flow cytometry. Box-and-whisker plots indicate median and minimum-maximum. MFI ratio, mean of fluorescence ratio.*


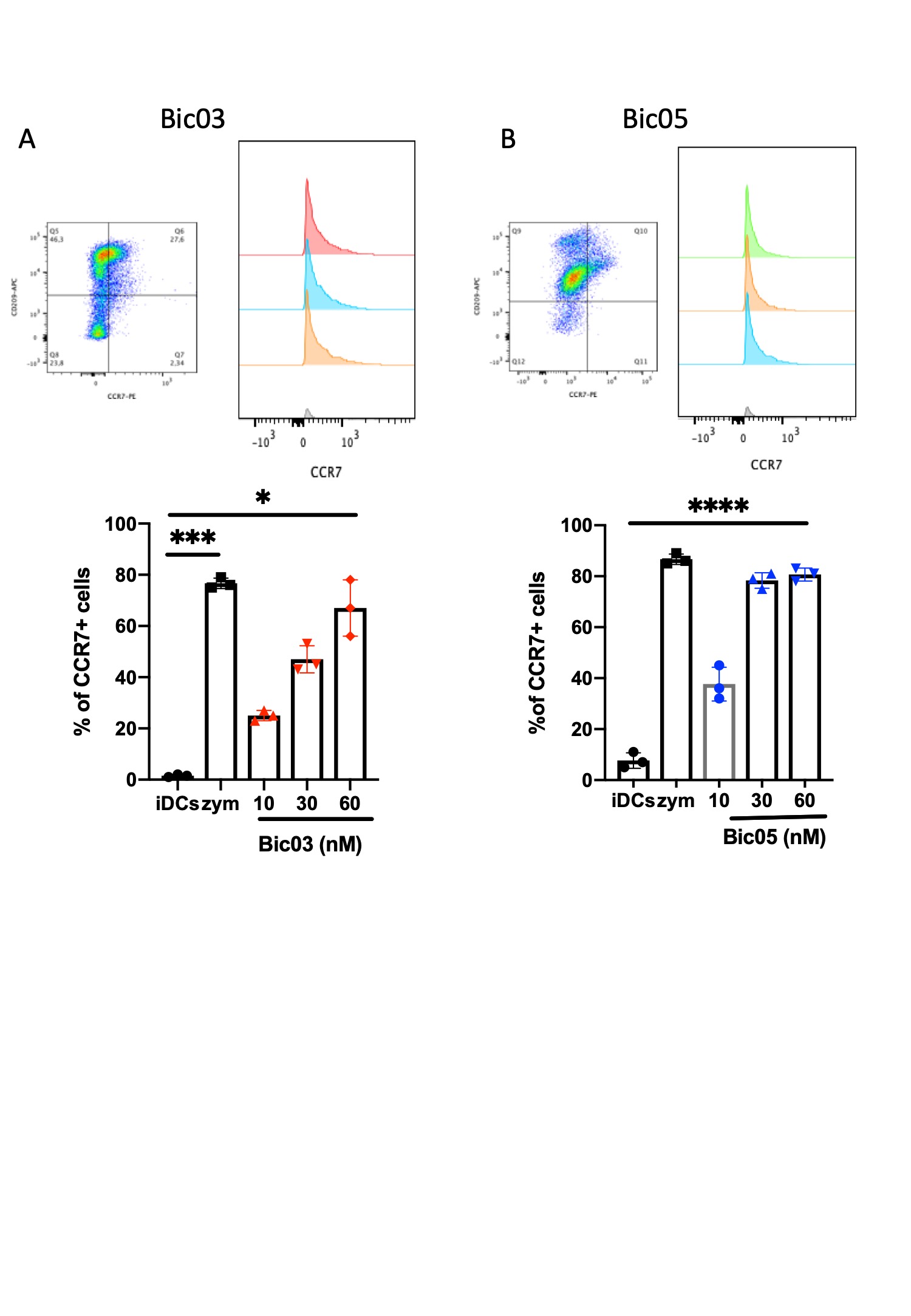


**Supplementary Figure 7.** ***Expression of CCR7 in Bic-treated moDCs****. (****A****) Bic03 and (****B****) Bic05 on flow cytometry (n=3, mean ± SD * p<0.01, *** p<0.001, ****p<0.0001).*


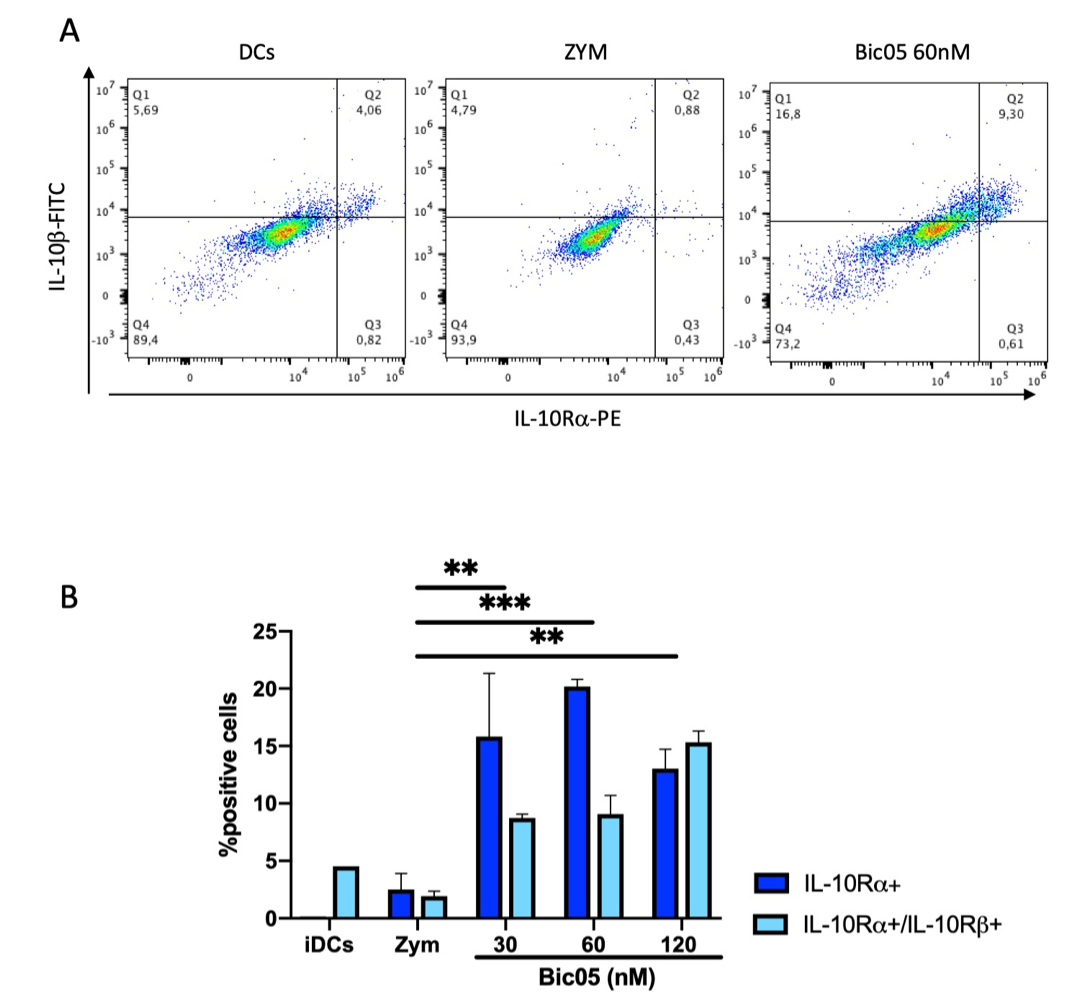


**Supplementary Figure 8.** ***Expression of IL-10 receptors on Bic05-treated moDCs.*** *(****A****) Dot plots of Bic05-treated* *moDCs on flow cytometry (1 of 4 experiments). (****B****) Expression of IL-10 Rα/Rβ receptors in the presence of Bic05 (n=4, mean ±SD, **p<0.01,***p>0.001).*


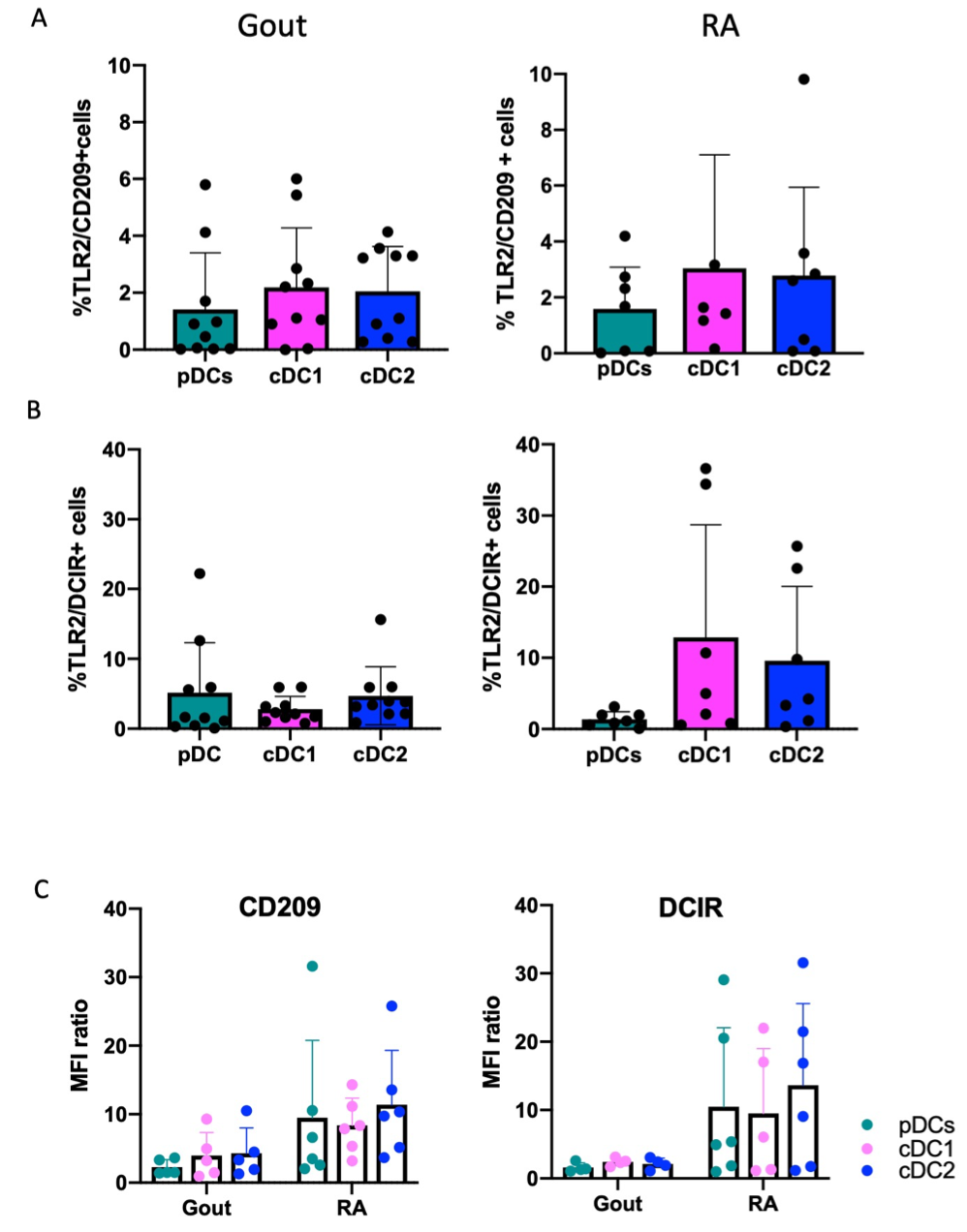


**Supplementary Figure 9**. ***SF DC phenotype***. *Percentage of DCs expressing (****A****) both TLR2 and CD209 and (****B****) both TLR2 and DCIR in total SF cells from gout and RA populations. (****A****) to (****B****) average % of n= 8 and 9 patients in gout and RA groups, respectively. (****C****) MFI ratio of CD209 and DCIR expression in DCs from gout and RA patients (n=6). SF, synovial fluid; RA, rheumatoid arthritis, MFI ratio, mean of fluorescence ratio.*

*
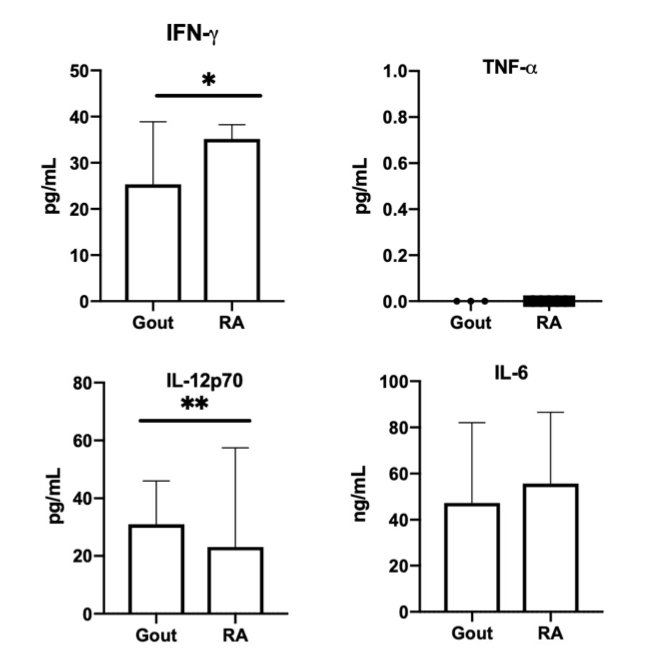
*

***Supplementary Figure 10****.* ***Cytokines in synovial fluid.*** *Measurement of cytokines (IFN-γ, TNF-α, IL-12p70, IL-6) in SF samples by ELISA (n=5, *p<0.06, **<p0.001). SF, synovial fluid.*

**Supplementary Table 1.**

NCT03416543

| **Characteristics** | **RA (n=9)** | **OA (n=8)** | **Gout (n=7)** |
| --- | --- | --- | --- |
| Age, years | 46 (20-81) | 69 (44-89) | 79 (49-89) |
| BMI, kg/m² | 26.6 (20-35) | 27.3 (22-28) | 31.2 (24-33) |
| Female sex, n (%) | 6 (75) | 5 (63) | 0 |
| Laboratory tests |  |  |  |
| WBC, ×10^6^/ml | 10.5 (6-18) | 5.5 (4-9) | 7.7 (7-11) |
| Lymphocytes, ×10^6^/ml | 1.9 (1-3) | 1.4 (1-2) | 1.4 (0.3-2) |
| CRP, mg/l | 28.4 (1-192) | 7.5 (5-105) | 150 (59-524) |
| Uric acid, µmol/l | 207 (161-246) | 365 (224-400) | 523 (172-872) |
| RF positivity, n (%) | 3 (33) | 1 (13) | 2 (29) |
| ACPA positivity, n (%) | 1 (11) | 0 | 0 |
| Values are presented as median (min-max) or n (%).  RA: rheumatoid arthritis, OA: osteoarthritis, BMI: body mass index; WBC : white blood cells; CRP: C-reactive protein; RF: rheumatoid factor | | | |

**Supplementary Table 1.** Baseline Characteristics of the Patients.
